# Automatic engagement estimation in smart education/learning settings: a systematic review of engagement definitions, datasets, and methods

**DOI:** 10.1186/s40561-022-00212-y

**Published:** 2022-11-12

**Authors:** Shofiyati Nur Karimah, Shinobu Hasegawa

**Affiliations:** 1 Graduate School of Advanced Science, Japan Advanced Institute of Science and Technology (JAIST), Nomi, Japan; 2 The Center for Innovative Distance Education and Research, Japan Advanced Institute of Science and Technology (JAIST), Nomi, Japan

**Keywords:** Engagement estimation, Engagement definitions, Engagement datasets, Engagement methods

## Abstract

**Background:**

Recognizing learners’ engagement during learning processes is important for providing personalized pedagogical support and preventing dropouts. As learning processes shift from traditional offline classrooms to distance learning, methods for automatically identifying engagement levels should be developed.

**Objective:**

This article aims to present a literature review of recent developments in automatic engagement estimation, including engagement definitions, datasets, and machine learning-based methods for automation estimation. The information, figures, and tables presented in this review aim at providing new researchers with insight on automatic engagement estimation to enhance smart learning with automatic engagement recognition methods.

**Methods:**

A literature search was carried out using Scopus, Mendeley references, the IEEE Xplore digital library, and ScienceDirect following the four phases of the Preferred Reporting Items for Systematic Reviews and Meta-Analyses (PRISMA): identification, screening, eligibility, and inclusion. The selected studies included research articles published between 2010 and 2022 that focused on three research questions (RQs) related to the engagement definitions, datasets, and methods used in the literature. The article selection excluded books, magazines, news articles, and posters.

**Results:**

Forty-seven articles were selected to address the RQs and discuss engagement definitions, datasets, and methods. First, we introduce a clear taxonomy that defines engagement according to different types and the components used to measure it. Guided by this taxonomy, we reviewed the engagement types defined in the selected articles, with emotional engagement (n = 40; 65.57%) measured by affective cues appearing most often (n = 38; 57.58%). Then, we reviewed engagement and engagement-related datasets in the literature, with most studies assessing engagement with external observations (n = 20; 43.48%) and self-reported measures (n = 9; 19.57%). Finally, we summarized machine learning (ML)-based methods, including deep learning, used in the literature.

**Conclusions:**

This review examines engagement definitions, datasets and ML-based methods from forty-seven selected articles. A taxonomy and three tables are presented to address three RQs and provide researchers in this field with guidance on enhancing smart learning with automatic engagement recognition. However, several key challenges remain, including cognitive and personalized engagement and ML issues that may affect real-world implementations.

## Introduction

Recognizing learners’ engagement can provide insight for enhancing learner-educator, learner-learning material, and learner-learner interactions (Sumer et al., 2021). Learner engagement has been found to be positively correlated with academic achievement (Lei et al., 2018), and higher engagement levels lead to better learning outcomes (Ponitz et al., 2009). A good engagement state is associated with curiosity, interest, optimism, and passion, which enhances motivation to continue learning and pursue achievement (Fredricks et al., 2004). Therefore, engagement is an essential component in the learning process that may reduce dropout rates, increase productivity and learning, and provide insights for improving course content and lecture plans (Alexander et al., 1997; Fredricks et al., 2004).

Research on engagement can be considered from two perspectives (Leite et al., 2015). Robot/computers/agents can be viewed as supports for increasing human engagement (Yun et al., 2012; Hall et al., 2014; Rich et al., 2010; Sanghvi et al., 2011) or as tools for automatically estimating human engagement (McDuff et al., 2012; Whitehill et al., 2014; Nakano and Ishii 2010; Castellano et al., 2009; Minsu et al., 2013; Castellano et al., 2012). In this article, we mainly focus on the second perspective.

Moreover, engagement estimation methods can be divided into three categories: manual, semiautomatic, and automatic (Dewan et al., 2019). In traditional offline classrooms, educators can recognize engagement levels directly or use manual observation checklists and rating scales. In contrast, in distance learning settings, learner engagement is more difficult to estimate due to limitations with learner-educator interactions. Therefore, a smart learning setting that allows automatic engagement estimation is one of potential solutions for addressing this limitation.

Recent improvements in computational hardware and software that support classic machine learning and deep neural networks have led to promising research on automatic engagement estimation (Gudi et al., 2015; Chaouachi et al., 2010). In particular, with the outbreak of severe acute respiratory syndrome coronavirus 2 (SARS-CoV-2), work in this field has increased considerably (Abdellaoui et al., 2020). Automatic engagement has become an important topic in several fields, such as human interaction research, including human-human interactions (HHIs) (Chatterjee et al., 2021), human-robot interactions (HRIs) (Yun et al., 2012; Rudovic et al., 2018b; Yue et al., 2019; Yun et al., 2020), human-computer interactions (HCIs) (Dubovi 2022; Monkaresi et al., 2017; Psaltis et al., 2018), and embodied conversational agents (ECAs) (AlZoubi et al., 2012). Furthermore, classroom applications are critical for improving smart education (Zaletelj and Košir 2017; Sumer et al., 2021; Ashwin and Guddeti 2020b).

Several automatic engagement estimation methods have been proposed in recent years. Among them, computer vision-based techniques are the most popular because nonverbal behaviours (including head motion, eye gaze, and body pose) play key roles in determining engagement levels (Ben-Youssef et al., 2019). In addition, computer vision-based approaches offer unobtrusive assessments, similar to classroom situations where teachers observe learners without interrupting their activities. These methods are also cost-effective and usable in the near term (D’Mello et al., 2017). Therefore, in this article, we conduct a systematic review on computer vision-based automatic engagement estimation methods that utilize appearance-based cues (such as videos or images).

Some physiological information-based methods that have received considerable attention in automatic engagement estimation research are also discussed. The development of cost-effective biosignal hardware, such as electroencephalogram (EEG), electrocardiogram (ECG), facial electromyogram (fEMG), and galvanic skin response (GSR), has provided simple and easy-to-use solutions (Alarcão and Fonseca 2019). Moreover, physiological signals support personalized analyses, which is pertinent for learners with special needs, such as those with autism (Rudovic et al., 2018b).

In this review, we aim to provide new researchers and educators in smart education and distance learning settings with an overview of the primary requirements and methods used to develop automatic engagement estimation methods, particularly in education/learning settings. The definitions, datasets, and methods are summarized in benchmark tables to provide an accessible overview of the systematic frameworks.

The research questions that guided this review were defined as follows:

- RQ1: How should the type of engagement to be measured be defined?
- RQ2: What datasets are suitable for developing automatic engagement estimation methods?
- RQ3: What automatic engagement estimation methods have been developed in the literature?

We followed the Preferred Reporting Items for Systematic Reviews and Meta-Analyses (PRISMA) to select articles for this review. In the literature, we first reviewed the definition of engagement to help new researchers understand what type of engagement they want to focus on. Understanding the type of engagement being focused on is important before engagement levels are measured to determine which engagement cues, datasets and methods should be used. The widely used datasets and machine learning-based methodologies for automatic engagement estimation are then examined.

The remainder of this article is organized as follows. The procedure for selecting the articles for this review is explained in “Review method” section. “Results and discussions” section presents the key finding based on the RQs. Finally, the conclusions, including the contributions, limitations, and future directions, are summarized in “Conclusion” section.

## Review method

The systematic review methodology employed in this study was adopted from the Preferred Reporting Items for Systematic Reviews and Meta-Analyses (PRISMA) model (Page et al., 2021). The review structure was guided from PRISMA 2020 Checklist addressing the abstract, introduction, methods, results, and discussion. However, for readability reason, we include the discussion in results section, which also is presented to address each RQ, and in conclusions section.

**Fig. 1:**
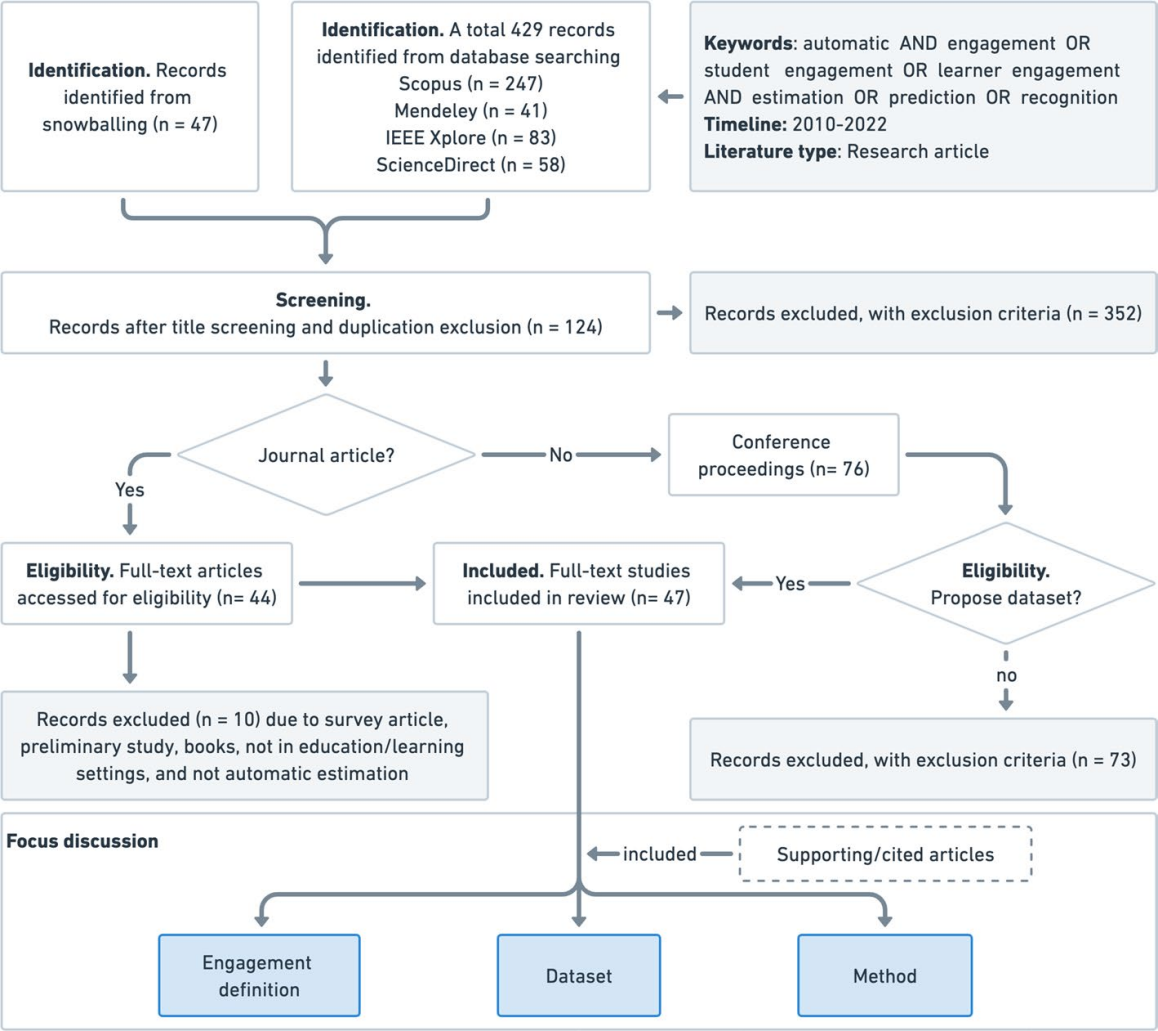
Illustration of the article selection process adopted from PRISMA flow diagram

A literature search was carried out based on PRISMA flow diagram with modification by adding eligibility phase. Therefore, there are 4 phases in the flow, i.e., identification, screening, eligibility, and inclusion. We also modify the flowchart by adding initial inclusion criteria (such as keywords, timeline, and literature type), and focus discussion (i.,e., engagement definition, dataset, and method). Figure 1 shows the modified PRISMA flowchart used to select articles in this review.

### Identification

The literature search was carried out by selecting research articles from the following electronic databases and libraries: Scopus, Mendeley, IEEE Xplore, and ScienceDirect. The following criteria were used to define the included studies:

- Focused on machine learning-based estimation
- Deployed in education/learning settings
- Journal publications or conference proceedings only if they developed an influential dataset for engagement estimation.

Based on the above criteria, we identified articles that satisfied the following terms: (1) keywords: automatic AND engagement OR student engagement OR learner engagement AND estimation OR prediction OR recognition; (2) publication year: 2010-2022; and (3) literature type: research article, excluding books, magazines, news articles, and posters. Additionally, to obtain more references, we used the snowballing approach by searching Google Scholar. A total of 429 articles were obtained in the identification phase according to the aforementioned search terms.

### Screening

In this phase, duplicate articles were excluded. Then, the titles and abstracts were scrutinized to determine whether they met the review criteria. The exclusion criteria included systematic reviews, surveys, and preliminary works (e.g., only report designs). With the exclusion criteria, 352 articles were excluded, yielding 124 articles.

### Eligibility

Journal articles and conference proceedings were assessed for eligibility in this phase. The titles, abstracts, main contents, and conclusions were examined to ensure that they met the inclusion criteria. In addition to the exclusion criteria mentioned in the screening phase, we also excluded articles that did not focus on automatic engagement estimation or were not related to education/learning settings. Even though face detection/recognition is a component of engagement estimation in some cases, we excluded articles that focused more on face detection/recognition than on engagement estimation.

A total of 10 journal articles were excluded in this phase according to the exclusion criteria. For the conference proceedings, only articles that proposed an influential dataset for engagement estimation were included. With this condition, 73 out of 76 articles were excluded.

### Inclusion

Finally, a total of 47 articles were selected, including 44 journal articles and 3 conference proceedings. In this review, we focused on three main topics: engagement definitions, datasets, and methods. In the discussion section, we also present some supporting articles with citations in the literature.

## Results and discussions

**Fig. 2:**
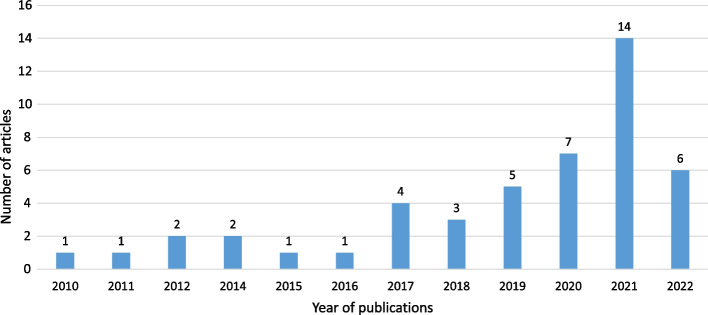
Number of reviewed articles per year

Forty-seven articles were selected for this review. The articles were published between 2010 and 2022, although no included articles were published in 2013. However, research on automatic engagement estimation in education/learning settings has increased in recent years (Fig. 2). In particular, in 2021, 14 articles (29.79%) on this topic were published (doubled from the previous year) following the outbreak of the COVID-19 pandemic, which started in 2020.

**Fig. 3:**
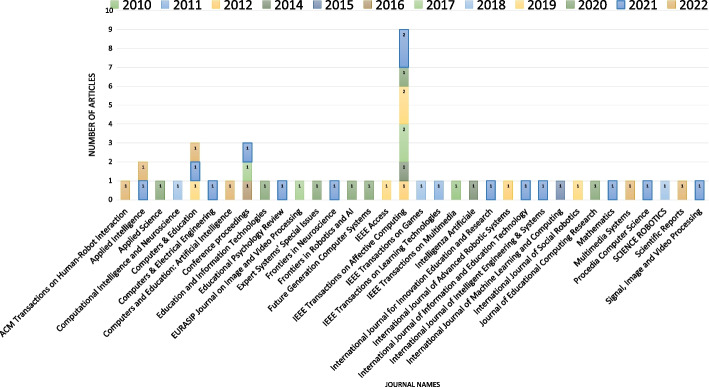
Number of reviewed articles per journal

Among the selected articles, 3 were published in conference proceedings, and the remainder were published in 33 different journals. Most of the journal articles were published in *IEEE Transactions on Affective Computing* ($n=9; 19.15\%$), *Computers & Education* ($n=3; 6.38\%$), and *Applied Intelligence* ($n=2; 4.26\%$), as shown in Fig. 3.

Interestingly, research on automatic engagement, which is aimed at education and learning settings, has been conducted in several research domains, including human-human interactions (HHIs), human-robot interactions (HRIs), human-computer interactions (HCIs), and embodied conversational agents (ECAs). From the reviewed articles, we also noted some studies based on data from offline classrooms. Therefore, we added the classroom as a separate research domain in this review.

**Fig. 4:**
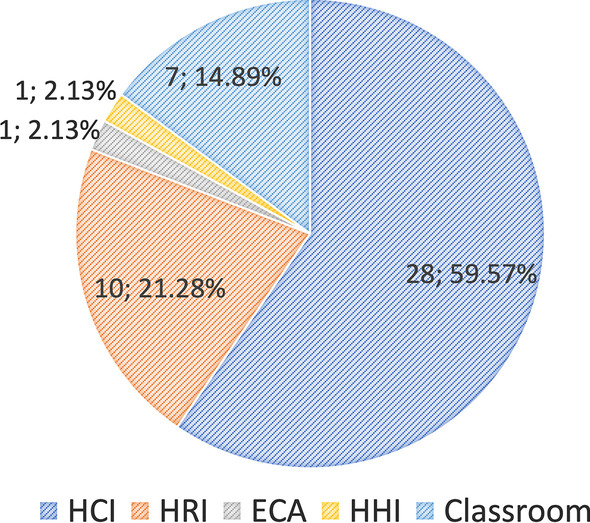
Number of reviewed articles per research domain

As shown in Fig. 4, research on automatic engagement estimation in education/learning settings was dominated by HCI ($n=28; 59.57\%$), followed by HRI ($n=10; 21.28\%$) and Classroom ($n=7; 14.89\%$) (see Appendix Table 2).

### RQ1: how should the type of engagement to be measured be defined?

In engagement estimation studies, the definition of engagement varies considerably. The definition of engagement depends on the main focus of the study (Christenson et al., 2012; Keen 2009). In educational or learning contexts, we found that the three main research domains depended on the engagement stimuli: HCIs, HRIs, and ECAs, HHIs.

Although HRI research is novel in the field of education, robots can assist humans in different learning processes, such as helping children learn cognitive and social skills and supporting educators teaching difficult concepts (Sharkawy 2021). Robots can not only assist in learning processes but can also measure and increase learner engagement (Celiktutan et al., 2019; Rudovic et al., 2018b; Del Duchetto et al., 2020). HRI researchers defined engagement via two approaches. The first approach defines engagement as a process during interactions that combines verbal and nonverbal communication between two (or more) partners, and the second approach defines engagement as an interaction quality metric.

Moreover, researchers who focused on ECAs (Peters et al., 2005) and intelligent tutor systems (ITSs) (D’Mello et al., 2007) viewed engagement as a value that indicates how likely a person is to remain with their partner and continue an interaction.

Furthermore, engagement estimation research in the field of HCI defined engagement based on engagement cues in computer-based learning, such as learners watching videos, writing, and playing educational games, or in classroom recordings (Whitehill et al., 2014; Monkaresi et al., 2017; Sumer et al., 2021).

**Fig. 5:**
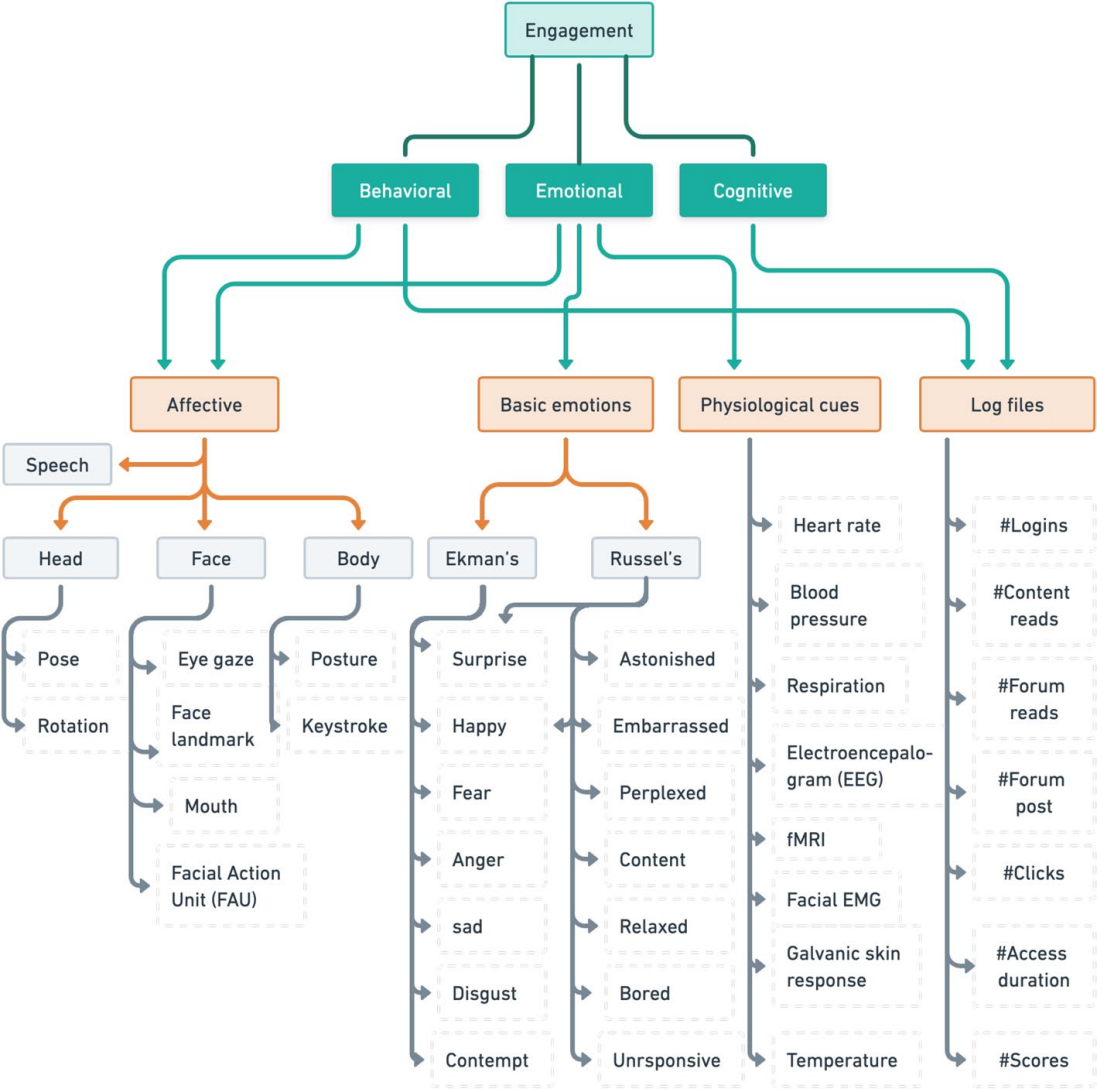
Taxonomy of engagement definition and its components

This inconsistent definition of engagement in the literature due to the lack of consensus and taxonomy for learning engagement (Yue et al., 2019) may cause confusion for new researchers in this field. To address this challenge, we introduce a taxonomy for engagement and systematically review the definition of engagement used in the selected articles (Fig. 5). As a baseline, we follow the definition of engagement in education and learning environments proposed by Fredricks et al., (2004), which has been widely used in engagement research (Wolters and Taylor 2012; Finn and Zimmer 2012; Greene 2015; Xie et al., 2019; Azevedo 2015).

Engagement is associated with internal states constructed by various cues and may not be visually apparent. Fredricks et al. (2004) divided engagement into three categories: behavioural, emotional, and cognitive engagement. Although, in this definition of engagement, the components to construct each type of engagement overlap considerably, as shown in Fig. 5.

*Behavioural* engagement describes learners’ participation in learning and tasks (Fredricks et al., 2004). In classroom settings, behavioural engagement is shown by actively participating in class, such as by asking questions or displaying attention and concentration (Sumer et al., 2021). *Emotional* engagement refers to learners’ affective reactions in the classroom or during learning, including interest, boredom, happiness, sadness, and anxiety (Fredricks et al., 2004). *Cognitive* engagement, which is also referred to as self-regulation, incorporates learners’ psychological investment in learning, including their flexibility in problem solving, learning motivation, and coping mechanisms when faced with failure.

The components for assessing engagement include effort, attention, and persistence for *behavioural* engagement; various emotional reactions (such as anger, surprise, disgust, enjoyment, fear, and sadness (Ekman and Friesen 1978)) to the learning materials for *emotional* engagement; and metacognitive strategies, namely, how learners set goals, plan, and organize their study efforts, for *cognitive* engagement (Fredricks et al., 2004).

In developing automatic engagement estimation methods, these components can be obtained with several modalities (Table 2), such as *log files*, which include information related to learner performance, reaction times, and errors (Cerezo et al., 2016; Okubo et al., 2017; You 2016); *affective cues*, including face and body analyses from video/images (Whitehill et al., 2014; Bosch et al., 2016; Bosch 2016); and *physiological cues*, such as galvanic skin responses (Di Lascio et al., 2018; McNeal et al., 2020), electroencephalograms (EEGs) (Poulsen et al., 2017; Bevilacqua et al., 2019), heart rates (Darnell and Krieg 2019; Monkaresi et al., 2017), and combinations of these cues (D’Mello et al., 2017).

The engagement level can be determined by grouping emotions according to Ekman’s basic emotions (Ekman and Friesen 1978) or Russel’s model (Russell 1980). For example, Altuwairqi et al., (2021a) suggested that ‘surprised’ indicates *strong* engagement; ‘enthusiastic’, ‘excited’, and ‘nervous’ indicate *high* engagement; ‘satisfied’ and ‘happy’ indicate *medium* engagement; and ‘bored’ indicates *low* engagement. Other behaviours, such as not looking at the computer and playing with hair, are classified as disengagement. For two-level classification, strong, high, and medium engagement are grouped into the high engagement class, while low and disengagement are grouped into the disengagement class. In addition, Olivetti et al., (2019) divided engagement level into three classes based on the first and fourth quadrants of Russell’s model: **Class 1** included bored, relaxed, and unresponsive; **Class 2** included happy, attentive, content, and perplexed; and **Class 3** included surprised, astonished, and embarrassed.

Consulting the taxonomy, we then reviewed the definition of engagement with a two-step approach. First, we examined the modalities used in each article and how the engagement level was determined. The articles included three common engagement modalities: affective cues (including audio and visual), physiological cues, and log files that were annotated to determine engagement. Some works used publicly available datasets or facial expression tools that already included basic emotion labels. Therefore, we included basic emotions in the taxonomy at the same level as the other modalities to further define the type of engagement (i.e., behavioural, emotional, or cognitive). Note that one engagement cue does not exclusively correspond to one engagement type, as previously discussed.

For example, Apicella et al. (2022) estimated emotional and cognitive engagement with a physiological sensor, i.e., EEG signal acquisition, because the type of stimuli considered during data collection was related to internal emotions and the cognitive task. In this case, two types of stimuli, namely, social feedback and background music, which were organized based on Russel’s four quadrants, were used to estimate emotional engagement, while a cognitive task (Continuous Performance Test) was used to estimate cognitive engagement.

Moreover, Goldberg et al. (2021) analysed three types of engagement with one modality, namely, videos recorded in an offline classroom. The behaviour of the students (on- or off-task) in the videos and a knowledge test presented during the lecture were used to estimate the behavioural and cognitive engagement levels, while facial features were extracted from the video to analyse emotional engagement. Therefore, in addition to the engagement cues used, defining what type of engagement is being measured depends on what stimuli were presented to the participant during data collection and what physical or cognitive behaviours were observed.

**Fig. 6:**
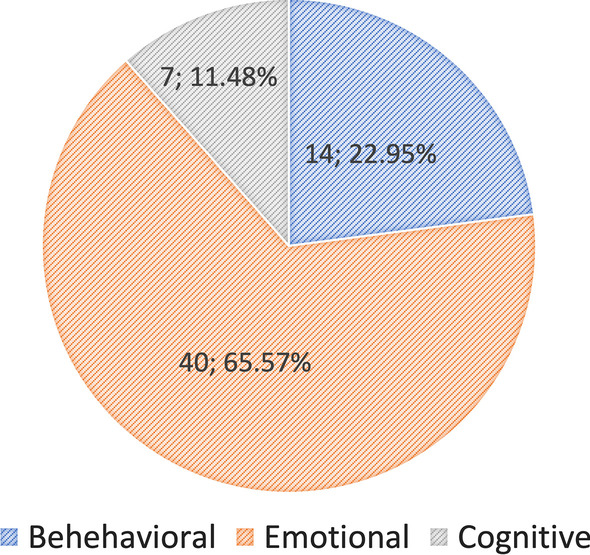
Pie chart of the engagement types estimated in the selected articles

**Fig. 7:**
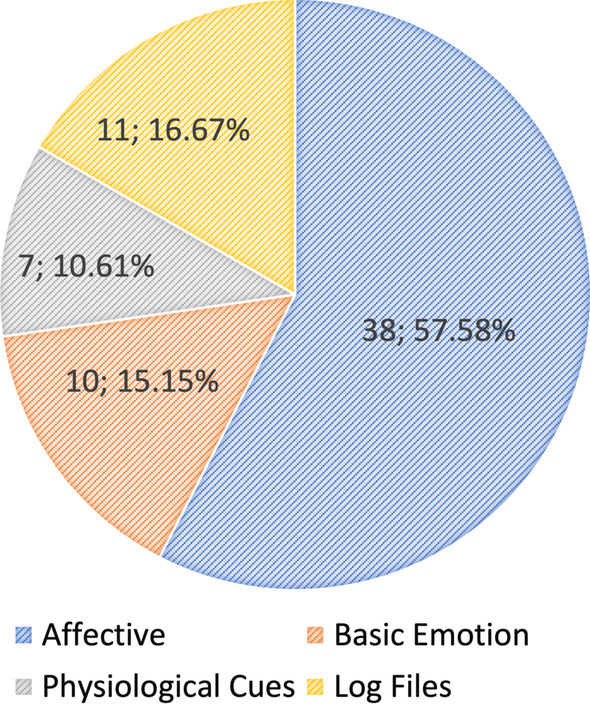
Pie chart of the engagement cues measured in the selected articles

Overall, most of the selected articles analysed emotional engagement ($n=40; 65.57\%$) with affective cues ($n=38; 57.58\%$), including visual (from videos, which show facial, body, and head information) and audio (speech) cues (Figs. 6 and 7) (See Appendix Table 2).

### RQ2: what datasets are suitable for developing automatic engagement estimation methods?

Adequately labelled data and a sufficient amount of data that includes as many generalized variables as possible are important criteria for developing automatic engagement estimation methods. Automatic engagement estimation approaches can be developed by using publicly available datasets or self-collected datasets. Publicly available datasets are open, freely downloadable and may have some terms and conditions, such as use in only research contexts or with consent from the authors. Moreover, self-collected datasets (also referred to as non-public datasets) are built according to specific tasks and cannot be publicly shared due to privacy policies and ethics.

In contrast to emotion recognition datasets, which are typically labelled based on Ekman’s basic expressions (e.g., anger, disgust, fear, happiness, sadness, surprise, and neutral), there are only a few publicly available engagement datasets, i.e., datasets that include ‘engagement’ in their labelling process. However, as shown in the taxonomy of engagement estimation (Fig. 5), an emotion recognition dataset can be used for automatic engagement estimation by modifying labels or by introducing other measurement metrics to define engagement types. In this article, we refer to datasets that are used in the automatic engagement estimation literature even though they have no straightforward engagement labels as *engagement-related datasets* and datasets that have ‘engagement’ label as *engagement datasets*.

The selected articles include four engagement-related datasets and three engagement datasets that are publicly available. The public engagement-related datasets include: (1) the NVIE dataset^1^ (Wang et al., 2010), (2) BAUM-1 (Zhalehpour et al., 2017), (3) the MASR dataset, which is used in Psaltis et al. (2016) but was proposed in Psaltis et al. (2016), and (4) AffectNet (Mollahosseini et al., 2019). The public engagement datasets include: (1) DAiSEE^2^ (Gupta et al., 2016), (2) UE-HRI^3^ (Ben-Youssef et al., 2017), and (3) MHHRI^4^ (Celiktutan et al., 2019) (see Appendix Table 3).

DAiSEE is one of the most popular publicly available engagement datasets used in the literature (Pabba and Kumar, 2022; Liao et al., 2021; Ma et al., 2021; Thiruthuvanathan et al., 2021; Mehta et al., 2022). Another popular publicly available engagement dataset is the Emotion Recognition in the Wild (EmotiW) dataset. This dataset was excluded from this review because the dataset is being continuously updated; however, EmotiW 2018 (Dhall et al., 2018) and 2020 (Dhall et al., 2020), are accessible for academic research (ACM International Conference on Multimodal Interaction 2020, 2020).

The data in DAiSEE and EmotiW were collected in ‘in-the-wild’ environments, where participants contributed to the data collection process by recording themselves showing their upper body while watching learning videos. The participants could join from anywhere, and no camera or lighting specifications were considered. Therefore, the quality (e.g., illumination, background noise, and occlusion) of the videos varies. Although in-the-wild data have considerable variations, they are believed to be the closest to real-world conditions (Gupta et al., 2016; Dhall et al., 2018, 2020).

Despite the ease and amount of available data, DAiSEE, EmotiW, and other publicly available datasets were collected with participants of certain ethnicities, which may not be appropriate for all target subjects. Moreover, ‘in-the-wild’ data may be difficult to process due to the large variations. Therefore, most engagement studies build custom engagement datasets that address the requirements of their model or system (Appendix Table 3). However, because data collection is costly and time-consuming, the amount of data collected may be insufficient. In such cases, self-collected data can be combined with engagement-related datasets or transfer learning data to enhance the estimation performance.

Transfer learning is a type of fine-tuning, which is briefly described in Sect. “Fine-Tuning and Transfer Learning Techniques”. In general, transfer learning involves using a pretrained neural network on a large dataset to extract features to use on tasks with smaller datasets. Some image datasets used for transfer learning include FER-2013 (Goodfellow et al., 2013), VGGFace (Parkhi et al., 2015), VGGFace2 (Cao et al., 2018), FaceNet (Schroff et al., 2015), AffectNet (Mollahosseini et al., 2019), 300W-LP and AFLW2000 (Zhu et al., 2016), JAFFE (Lyons et al., 2002), CK+ (Lucey et al., 2011), and RAF-DB (Li et al., 2017) (see Appendix Table 3).

#### Engagement measurement

**Fig. 8:**
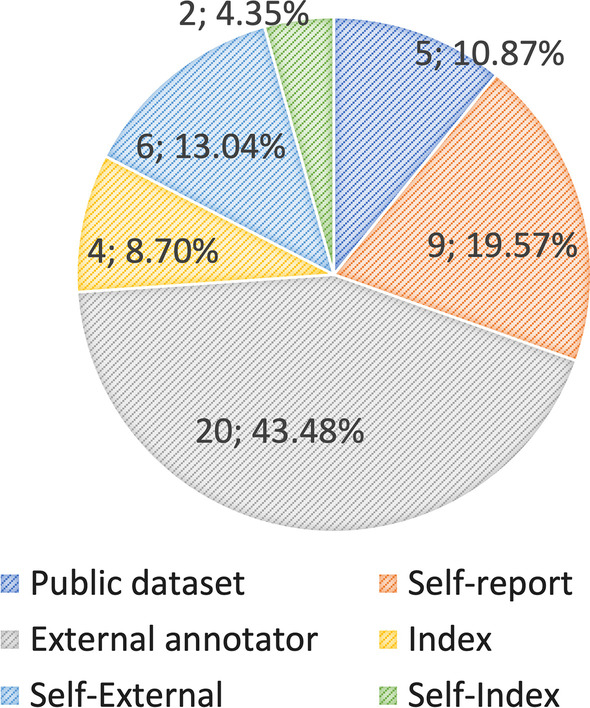
Pie chart of the engagement measurements and annotations used in the selected articles

There are various approaches for measuring engagement, including self-reports, experience sampling techniques, teacher ratings, interviews, and observations (Fredricks and McColskey 2012). In addition, different indices (such as performance indices, number of clicks, and sensor data) have been used to assess engagement (Yue et al., 2019; Apicella et al., 2022; Yun et al., 2012). However, external observations, self-reported measures and ratings are commonly used to measure engagement (Whitehill et al., 2014; Christenson et al., 2012). Moreover, most publicly available engagement datasets were collected based on external observations by external annotators (*n* = 20; 43.48%) (Fig. 8).

Self-reported measures are cheaper and easier to collect than external observations, which require more personnel to measure engagement (Christenson et al., 2012). Self-reports can be performed by self-annotating or completing questionnaires related to self-engagement (O’Brien and Toms 2010). However, self-reported measures are prone to Dunning-Kruger effects, as people are biased in recognizing self-competence (Kruger and Dunning 1999; Pennycook et al., 2017). In addition, these measures dependent strongly on participant compliance and diligence (Eisele et al., 2022). The bias associated with self-reported measure was also observed by Ramanarayanan et al. (2017b; a).

Furthermore, observational measures limit the judgement quality of learners’ actual effort, participation, or thinking (Fredricks et al., 2004; Peterson et al., 1984). An external observer is an overhearer (Schober and Clark 1989) that may not consider nonverbal behaviours as signs of engagement. For example, learners who are judged to be on-task or engaged by observers may not actually be thinking about the learning material. In contrast, some learners who appear to be off-task or unengaged may be attempting to understand or relate new ideas to what they have learned (Peterson et al., 1984). In addition, in terms of cognitive engagement, cognition is not easily observable and must be inferred from behaviours or assessed according to performance or self-reported measures (Fredricks et al., 2004; Winne and Perry 2000).

Alternatively, index measurements and combination approaches have been applied to reduce bias. Among the selected articles, four $(8.70\%)$ studies used index measurements, six $(13.04\%)$ studies combined self-reported measures with external observations, and two $(4.35\%)$ studies combined self-reported measures with some index.

Trindade et al. (2021) performed calculations on log data from courses in Moodle to evaluate engagement. Similarly, Hasnine et al. (2021) calculated concentration indices, Apicella et al. (2022) combined self-reported measures with performance indices, and Yue et al. (2019) combined self-reported measures with quiz scores to assess engagement.

#### Annotations

Annotation is a crucial step in building a good dataset. Single data points can be annotated manually by one or multiple annotators or by using a framework (Chi and Wylie 2014) or annotation tools such as CARMA (Girard 2014), ANVIL (Kipp 2008), NOVA (Baur et al., 2015), and ELAN (Wittenburg et al., 2006; Brugman and Russel 2004), as shown in Table 3.

To determine whether the labels are consistent, an agreed-upon final label must be determined by several annotators, for example, by using Cohen’s kappa value (Wang et al., 2010; AlZoubi et al., 2012; Whitehill et al., 2014; Zhalehpour et al., 2017; Ashwin and Guddeti 2020a, b). Cohen’s kappa has also been used to evaluate the efficiency of classifiers for multiclass and imbalanced data (Thiruthuvanathan et al., 2021).

The final label can also be determined by measuring intraclass correlations (ICCs) (Goldberg et al., 2021; Rudovic et al., 2018b) or by applying the majority-vote aggregation technique (Yun et al., 2020; Pabba and Kumar 2022; Zhalehpour et al., 2017). Highly consistent labelled data usually indicate a high degree of credibility (Zhang et al., 2020).

#### Labelling issues

Visual computer vision-based engagement estimation datasets encounter several challenges, such as various camera angles and image quality (illumination, background, occlusion, etc.). In addition, the difficulty in capturing subtle changes in visual appearance leads to mislabelling issues. For example, one video clip may show more than one engagement state annotated as one state. As a result, some frames may be mislabelled, potentially influencing the frame-by-frame estimation process (Yun et al., 2020). Frame-based labelling is viewed as the easiest solution. However, this approach lacks continuous labels, which provide more precise information (Sumer et al., 2021). To address this issue, temporal dynamics features need to be extracted (Yun et al., 2020).

However, in some cases, some frames are more significant for determining engagement levels, while other frames can mislead the final estimation result (Zhu et al., 2020). One solution for addressing this problem is applying an attention mechanism. The attention mechanism in deep learning directs attention to effectively choose important frames (Vaswani et al., 2017; Winata et al., 2018).

Another labelling issue is false interpretation. For example, learners may be engaged regardless of where they are looking, and observers might label a learner who looks down as disengaged while the learner is actually thinking or processing the learning material. Especially in higher grade levels, learners may show/hide their engagement, and engagement cues may thus be more difficult to identify (Lufi and Haimov 2019). Moreover, age can affect attention levels (Lufi and Haimov 2019). Therefore, collecting an engagement dataset that represents learners’ authentic internal states is a challenging task.

### RQ3: what automatic engagement estimation methods have been developed in the literature?

Machine learning, which is a subset of artificial intelligence (AI), is known for its capability to acquire knowledge to make decisions by extracting patterns from raw data (Goodfellow et al., 2016). Machine learning techniques have been applied in various fields, including agriculture, transportation, business, and education. Machine learning has led to the development of affective computing methods that automatically recognize human emotions and behaviours (Schuller 2015; Kratzwald et al., 2018; Zhao et al., 2019; Rouast et al., 2021), supporting the advancement of artificial intelligence in education applications (Chen et al., 2020; Ouyang and Jiao 2021). Therefore, in general, automatic engagement estimation methods are referred to as machine learning (ML)-based algorithms.

**Fig. 9:**
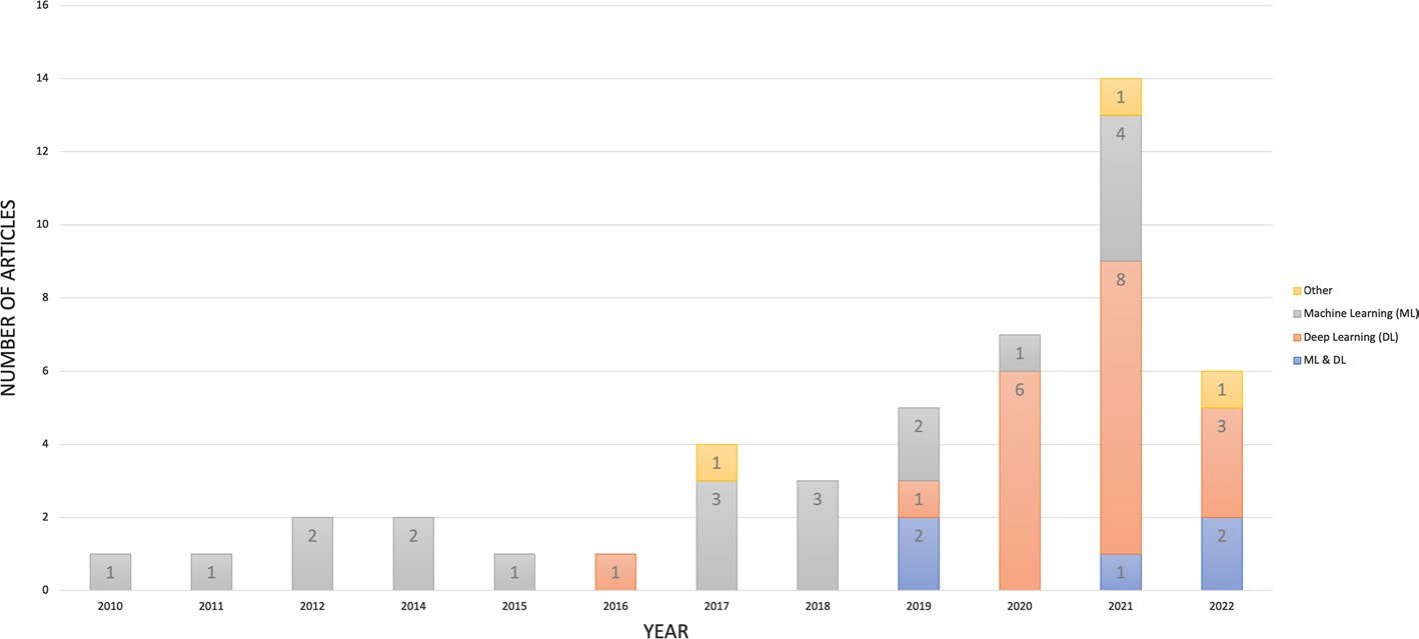
The general method used in the selected articles

Since machine and deep learning methods are the most commonly used approaches for developing automatic engagement estimation tools in the literature (Fig. 9), in this section, we briefly discuss the pre-processing steps and estimation methods (classification or regression). We classified the estimation methods as classic machine learning and deep learning techniques.

**Fig. 10:**
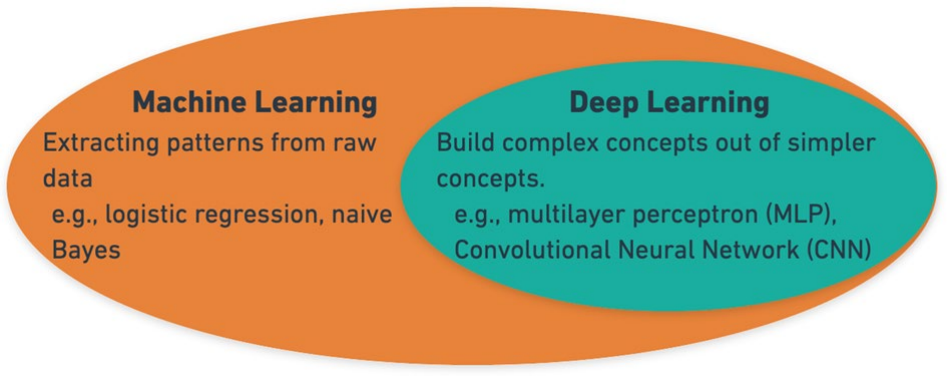
The difference between machine learning and deep learning

Deep learning is a subset of machine learning. Both techniques work by mapping raw data features to extract the desired information. Nevertheless, it may be difficult for computers to extract features from raw data with large variations, and these features may be identifiable only using a nearly human-level understanding of data (Goodfellow et al., 2016). Therefore, classic machine learning methods require hand-designed features. Moreover, deep learning approaches reduce the desired complicated mapping into a series of nested mappings that can be described by layers (Goodfellow et al., 2016). For example, to identify image features, the input is presented as a visible layer. Then, the next layers, namely, the hidden layers, divide the image into smaller maps such as edges, corners and contours, object parts, and finally, the object identity. Figure 10 depicts a Venn diagram showing how deep learning is distinguished from classic machine learning.

#### Pre-processing

Before data can be fed into a network, the raw data must be pre-processed to extract the features. Video/image-based data can be pre-processed with face detection, tracking, and cropping techniques (Yun et al., 2020). Alternatively, statistical values can be extracted to obtain representation information from features in a given time window (Hernandez et al., 2013; Sanghvi et al., 2011; Yun et al., 2020). Statistical rules such as sum, max, min, and mode can be utilized to aggregate meaningful information as input for classifiers, including support vector machines (SVMs) and neural networks (Yun et al., 2020).

*Face detection and feature extraction* Appearance-based features can be divided into two categories: low-level features and high-level features. Low-level features include the information generated in each video frame in a given time window. In particular, HCI engagement research has adopted low-dimensional geometry and appearance descriptors as features (Sumer et al., 2021; Whitehill et al., 2014). Additional low-level features include local binary patterns in three orthogonal planes (LBP-TOP), Gabor features, and box filters (BFs) (Li and Deng 2020).

High-level features are features extracted by aggregating low-level features (Yun et al., 2020), such as facial action units (FAUs) and head poses. Facial features and head poses are some of the most commonly used features for determining engagement and attention (Akker et al., 2009; Ba and Odobez 2006; Dong et al., 2010; Voit and Stiefelhagen 2008; Zhang et al., 2007). These features can be extracted statistically or by using facial recognition tools, as shown in Table 1.

**Table 1:** Face recognition tools for face detection and feature extraction

Tools name	Used in
OpenFace (Baltrusaitis et al. 2016, 2018)	(Kaur et al. 2018; Rudovic et al. 2018b; Goldberg et al. 2021; Ma et al. 2021; Wu et al. 2020; Zhang et al. 2019; Zhu et al. 2020; Thong Huynh et al. 2019; Li et al. 2021; Engwall et al. 2022)
OpenCV	(Yang et al. 2018; Wang et al. 2020; De Carolis et al. 2019; Bhardwaj et al. 2021; Hasnine et al. 2021)
Dlib	(Hasnine et al. 2021; Mehta et al. 2022)
OpenPose (Cao et al. 2017)	(Vanneste et al. 2021; Zheng et al. 2021; Wu et al. 2020; Zhu et al. 2020)
RetinaFace (Deng et al. 2019)	(Sumer et al. 2021)
FasterRCNN (Ren et al. 2015)	(Rudovic et al. 2018a)
faceAPI	(Castellano et al. 2012)
Affectiva API in iMotion	(Dubovi 2022)

OpenFace is a popular computer vision toolkit for extracting facial features, including in automatic engagement estimation research (Table 1). OpenFace implements multitask cascaded convolutional networks (MTCNNs) (Zhang et al. 2016) for face detection, constrained local models (Baltrusaitis et al., 2013; Zadeh et al., 2017) for landmark detection and tracking, eye rendering (Wood et al., 2015) for eye gaze estimation, and cross-dataset learning and person-specific normalisation for facial action unit (FAU) detection. In addition, the OpenCV^5^ face detection library (Haar Cascade (Viola and Jones 2004, 2001; Schmidt and Kasiński 2007)) and Dlib library for face and landmark detection are widely used. The mean shift-based object tracker in OpenCV can also be used for face tracking. Furthermore, in HRI, face recognition can be performed by utilizing the software development kit (SDK) built into the robot, for example, the NAOqi People Perception in the Pepper robot (Ben-Youssef et al., 2021). Interested readers are referred to (Wang and Deng 2021) for an in-depth explanation, especially deep learning-based face recognition.

*Data augmentation* Data augmentation is the process of creating new data based on real data without changing the original data. For image inputs, data augmentation can be performed by flipping (horizontally or vertically), cropping, scaling, or translating/rotating the images. As a result, the sampling rate for the input can be increased by adding the augmented data to the original dataset (Shen et al., 2022; Ashwin and Guddeti 2020b; Pabba and Kumar 2022).

*Feature selection* Feature selection not only determines the optimal set of features but also ranks and compares the most discriminative features. Some feature selection methods include F-scores (Chen and Lin 2006), RELIEF-F (Whitehill et al., 2014), DeepLift (Rudovic et al., 2018b), and recursive feature elimination random forests (RFE-RFs). Alternatively, ANOVA can be used to analyse the significance of labelled features (Schiavo et al., 2014).

*Dimensional reduction* Dimensional reduction is the process of decreasing the dimension of the input feature to prevent overfitting (Yun et al., 2020). Dimensional reduction can be applied to a dataset before the data are fed into the network. Some dimensional reduction methods include principal component analysis (PCA) (Sumer et al., 2021; Wang et al., 2010) and forward feature selection (FFS) (AlZoubi et al., 2012). However, dimensional reduction can also be performed by layer reduction using various pooling layers (max, average, and variance pooling, 1x1 convolutional layers) when a convolutional neural network is utilized (Yun et al., 2020).

*Addressing imbalanced data* One major issue with engagement datasets is imbalanced data that are severely skewed towards the majority class (Yun et al., 2020). Imbalanced class labels often occur because disengagement is rarely observed in continuous labelling. Many methods have been proposed to address this issue (Galar et al., 2012; Chawla et al., 2002; García et al., 2012; Dresvyanskiy et al., 2021). There are three categories of resampling techniques (Ben-Youssef et al., 2021): (1) undersampling methods, which aim to balance class distributions by eliminating majority class examples; (2) oversampling methods, which generate minority class examples, e.g., the synthetic minority oversampling technique (SMOTE) (Chawla et al., 2002); and (3) hybrid methods that combine both sampling methods (García et al., 2012; Chawla et al., 2002). Moreover, continuous scales may be discretized into groups (Rudovic et al., 2019a, b), and weighting techniques (Dresvyanskiy et al., 2021; Lin et al., 2017) have also been used to address this problem.

#### Classic machine learning methods

Engagement is estimated by calculating probabilities. To calculate the engagement probability, several classic machine learning methods can be utilized, such as the support vector machine (SVM) and its variations (including support vector regression (SVR)), naive Bayes (NB), decision trees (DTs), logistic regression (LR), clustering techniques (e.g., K-nearest neighbour (KNN)), and random forest (RF). These machine learning techniques are conveniently available in machine learning toolboxes such as Waikato Environment for Knowledge Analysis (WEKA) (Witten and Frank 2005) (as used in (Cocea & Weibelzahl 2011; Monkaresi et al., 2017; Ribeiro Trindade and James Ferreira 2021)), the Computer Expression Recognition Toolbox (CERT) (Littlewort et al., 2011) (as used in (Whitehill et al., 2014)), and the MATLAB library ((Chatterjee et al., 2021; AlZoubi et al., 2012).

**Fig. 11:**
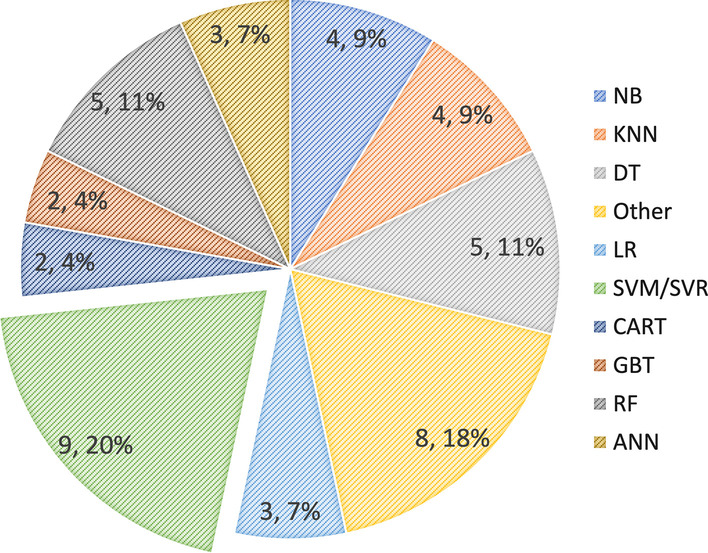
Pie chart of the use of classic machine learning methods for automatic engagement estimation

Between 2010 and 2022, classic machine learning methods dominated the automatic engagement estimation literature (Fig. 9), especially SVMs (Fig. 11). Note that some of the selected articles examined more than one algorithm. Therefore, the totals in Fig.11 do not correspond to the number of selected articles (see Appendix Table 4).

#### Deep learning methods

With the development of deep learning, research on automatic engagement estimation has applied these techniques to improve the estimation performance (Fig. 9). In this section, we briefly introduce some deep learning methods, including those used in the selected articles. For a more detailed explanation on deep learning techniques (especially for face recognition), interested readers are referred to (Wang & Deng, 2021; Fuad et al., 2021; Li & Deng, 2020).

**Fig. 12:**
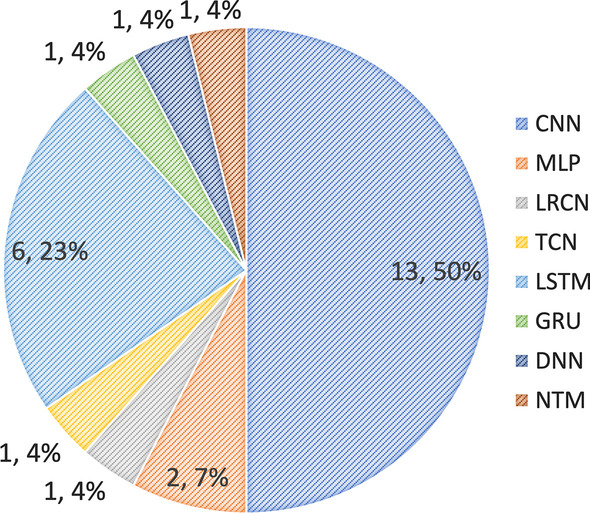
Pie chart of the use of deep learning methods for automatic engagement estimation

*Multilayer perceptron (MLP)* The multilayer perceptron (MLP), also called the feedforward neural network or deep forward network, was one of the first deep learning algorithms. The MLP is a mathematical function that is formed by combining many simpler functions to map some set of input values to output values (Goodfellow et al., 2016). An MLP consists of at least three layers of nodes, i.e., the input $f^{(1)}$, hidden $f^{(2)}$, and output $f^{(3)}$ layers, to define the mapping $y \approx f^*({\textbf {x}}) = f({\textbf {x}}) = f^{(3)}(f^{(2)}(f^{(1)}({\textbf {x}})))$. The first and last layers are called the input and output layers, respectively, while the number of hidden layers may be varied, which determines the *depth* of the model. Furthermore, each layer may contain more than one unit depending on the number of inputs and outputs. This algorithm has also been used in automatic engagement estimation for performance comparison with other algorithms (Ben-Youssef et al., 2019; Sumer et al., 2021; Rudovic et al., 2018b).

*Convolutional neural network (CNN)* A convolutional neural network (CNN) is a specialized kind of deep learning (DL) algorithm for processing data that employs mathematical linear operations known as *convolutions* as opposed to matrix multiplication (Goodfellow et al., 2016). The convolution operation is typically denoted with an asterisk: $x'(t) = (x * w)(t)$, where $x'$ is the *feature map*, i.e., the estimated value from the convolution of the *input* *x* with a *kernel* *w* at time *t* (Goodfellow et al., 2016).

CNNs are currently one of the most popular methods in different fields (Fig. 12). This technique has been widely used in various computer vision applications, including image classification (He et al., 2016), semantic segmentation (Noh et al., 2015), object detection (Szegedy et al., 2015), face recognition (Parkhi et al., 2015), spatiotemporal feature learning (Tran et al., 2015; Husain et al., 2016; Ji et al., 2013; Yun et al., 2020; Rudovic et al., 2018a; Abedi and Khan 2021; Ashwin and Guddeti 2020c; Yue et al., 2019), and automatic engagement estimation (see Appendix Table 4).

CNNs are popular because they can be highly modified and pretrained. Some CNNs include AlexNet (Krizhevsky et al., 2017), i3D (Carreira and Zisserman 2017), VGG16 (Simonyan and Zisserman, 2014), and ResNet (He et al., 2016; Szegedy et al., 2015).

The inputs to a CNN are usually greyscale or RGB images. The use of multiple small filtering kernels allows the network to extract more discriminative features because multiple small kernels are easier to optimize than one large filter kernel (Mohamad et al., 2020; Wang et al., 2020). However, CNNs have some crucial issues, such as large training times, gradient vanishing due to the use of deep networks, and a large number of parameters (Thiruthuvanathan et al., 2021).

*Recurrent neural network (RNN)* A facial expression changes through three stages, i.e., onset, apex, and offset (Liu et al., 2014). In recurrent neural network (RNN) algorithms for engagement estimation, time-series images are more reasonable than static images as input since time-series present sequence-related task information (Jordan 1990). RNNs capture information at earlier and later time steps by remembering each piece of information over time (Sharkawy 2020). Therefore, this algorithm has become a more popular automatic engagement estimation method (see Appendix Table 4).

Some types of RNNs include long short-term memory (LSTM) (Hochreiter and Schmidhuber 1997) (Yue et al., 2019; Ben-Youssef et al., 2019; Del Duchetto et al., 2020; Liao et al., 2021; Sumer et al., 2021; Engwall et al., 2022), gated recurrent units (GRUs) (Ben-Youssef et al., 2019), and network Turing machines (NTMs) (Qiao and Bi 2020; Ma et al., 2021).

However, despite advantages such as considerable computational power in temporal processing models and applications, in practice, RNNs are difficult to train due to network instability (Sharkawy 2020). Moreover, the networks may suffer from short-term memory issues if the input sequences are too long. Thus, RNNs may have difficulty capturing earlier time step information due to vanishing gradients (Sharkawy 2020).

Therefore, the attention mechanism was introduced to learn to associate the elements in sequence *C* with the elements in the output sequence (Bahdanau et al., 2014). The attention mechanism essentially determines a weighted average that is used to focus on specific parts of the input sequence at each time step (Goodfellow et al., 2016). Although the attention mechanism was originally introduced in the context of machine translation (Bahdanau et al., 2014), it has also been utilized in DL applications for automatic engagement estimation (Liao et al., 2021; Sumer et al., 2021; Mehta et al., 2022; Shen et al., 2022).

*Other classifiers* Other neural network techniques that have been used for automatic engagement estimation include the fuzzy min-max neural network (FMMNN) classifier (Simpson 1992; Gabrys and Bargiela 2000), which was implemented by (Yun et al., 2012) for automatic engagement estimation, the deep belief network (Hinton et al., 2006), which was used in (Dewan et al., 2018), and linear discriminant analysis (LDA) (Apicella et al., 2022; Wang et al., 2010).

#### Fine-tuning and transfer learning techniques

One fine-tuning technique for addressing insufficient training data is applying transfer learning, which utilizes networks pretrained on a large number of images (Bengio 2011; Wang and Deng 2021). Various models have been trained on large face image datasets. For example, Sumer et al. (2021) used AffectNet (Mollahosseini et al., 2019) and 300W-LP (Zhu et al., 2016), which were trained on ResNet50, for transfer learning. The pretrained models help the engagement estimation network learn general features related to face identification (Yun et al., 2020). As mentioned in Sect. “RQ2”, other large datasets that have been used for transfer learning include FER-2013 (Goodfellow et al., 2013), VGGFace (Parkhi et al., 2015), VGGFace2 (Cao et al., 2018), FaceNet (Schroff et al., 2015), AffectNet (Mollahosseini et al., 2019), 300W-LP and AFLW2000 (Zhu et al., 2016).

#### Performance metrics

To judge the automatic engagement estimation performance, the prediction results should be compared with human judgements in the dataset (Whitehill et al., 2014; Yun et al., 2020). In machine learning pipeline, performance metrics are used to monitor and measure the performance of a model depend on the task. Automatic engagement estimation problem can be seen either as classification or regression task. An engagement estimation is a classification task if the engagement is estimated in discreet class, e.g., low engagement class vs high engagement class. Otherwise, an engagement estimation is a regression task when continuous output desired. Some metrics used to measure the performance of regression task are Mean Absolute Error (MAE), Mean Squared Error (MSE), and Root Mean Squared Error (RMSE), which mainly calculating the distance between the predicted and the ground truth.

Classification performance metrics evaluate the estimation model that compares discrete classes, such as accuracy, precision and recall, F1-score, and Area Under the Curve-Receiver Operating Characteristics (AUC-ROC). Moreover, confusion matrix is also used to visualize the ground-truth labels versus the predicted results in a table.

Accuracy metric defines the number of correct predictions (true positive (TP)) divided by the total number of predictions. It is the most common metric for evaluating classification performance due to its simplicity. However, Accuracy may not be reliable when the dataset is severely unbalanced. In a severely skewed dataset, the classifier may not discriminate well despite high accuracy values because the classifier identifies only the most common class.

Alternatively, Precision/Recall (PR) trade-off curve (used in (Leite et al., 2015)) and F1-score (Schiavo et al., 2014) are used to overcome the limitation of Accuracy. Precision determines the performance by calculating the proportion of TP prediction to the total positive prediction (TP + false positive (FP)). Similarly, Recall calculates the TP prediction to the total number of TP and false negative (FN). Meanwhile, F1-score is the harmonic mean between the precision and recall.

Some alternative metrics that are more informative and “imbalance-friendly” include the balanced accuracy, AUC-ROC (Hernandez et al., 2013; Leite et al., 2015) and 2-alternative forced choice (2AFC) (Whitehill et al., 2014).

**Fig. 13:**
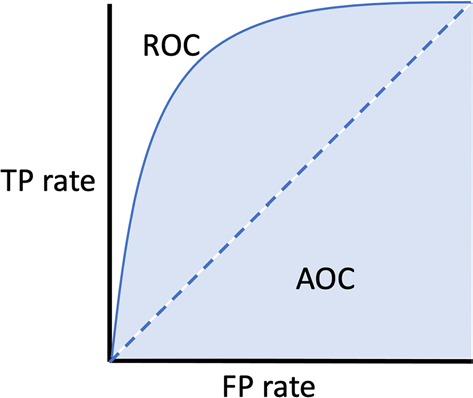
AUC-ROC curve illustration

AUC-ROC visualizes the classification performance based on correct and incorrect classifications (Fig. 13). The ROC curve plotted the trade-off between the TP rate (Recall) to the FP rate. AUC represents the degree or measure of separability between classes as a summary of the ROC curve (Bradley 1997). The AUC scores between $0.7-0.8$, $0.8-0.9$, and $>0.9$ are considered acceptable, excellent, and outstanding, respectively (Mandrekar 2010; Li et al., 2021).

$$\begin{aligned}{} & {} Precision = \dfrac{TP}{(TP + FP)} \quad \quad Recall\;(TP \text { rate}) = \dfrac{TP}{TP + FN} \\{} & {} FP \text { rate} = 1 - TP \text { rate} = \dfrac{FP}{TN + FP} \quad \quad F1-Score = 2 * \dfrac{Precision * Recall}{Precision + Recall} \end{aligned}$$

The 2-alternative forced choice (2AFC) (Mason & Weigel ,^2009^; Tingfan et al., 2012) is an unbiased estimate of the AUC-ROC curve since it expresses the probability of discriminating true positives (TP) from true negatives (TN). A 2AFC value of 1 indicates perfect discrimination, while a value of 0.5 indicates that the classifier performs at chance levels.

Furthermore, other metrics such as Matthews correlation coefficient (MCC) (Tingfan et al., 2012) and specificity and sensitivity (Yun et al., 2020) are also used in the engagement estimation literature. (see Appendix Table 4).

## Conclusion

This article reviewed recent research on automatic engagement estimation in education/learning settings, focusing on work published between 2010 and 2022. In particular, this review examined engagement definitions, datasets, and machine learning-based methods from forty-seven selected articles. The article selection and review methodology were adopted from the Preferred Reporting Items for Systematic Reviews and Meta-Analyses (PRISMA) model (Page et al., 2021) to answer three research questions:

- RQ1: how should the type of engagement to be measured be defined?
- RQ2: what datasets are suitable for developing automatic engagement estimation methods?
- RQ3: what automatic engagement estimation methods have been developed in the literature?

The results and discussion with the presented information, figures, and tables aim at providing new researchers with insight on automatic engagement estimation to enhance smart learning with automatic engagement recognition methods.

To answer the RQ1, we examined the definitions of engagement used in the selected articles and introduced an engagement definition taxonomy (Fig. 5) as a guide for educators and engagement estimation research, particularly for education/learning purposes. The taxonomy defined three types of engagement: behavioural engagement, emotional engagement, and cognitive engagement. Each engagement type was connected with some engagement cues, including affective cues, physiological cues, log files, and basic emotions. The modalities for obtaining engagement cues were also discussed, including speech cues, visual cues (face, head, and eye gaze), physiological sensor data, and log data.

From the discussion, we found that to define what type of engagement is being measured depends on engagement cues used, what stimulies presented to the participant during data collection, and what physical or cognitive behaviours observed. We believe that the proposed taxonomy will allow for enhanced research on automatic engagement estimation.

The datasets used in the literature were summarized in this review to address the RQ2. The datasets include publicly available datasets and self-collected datasets. In this review, publicly available datasets were divided into two categories, namely, engagement datasets and engagement-related datasets, to distinguish the availability of engagement labels. The engagement measurement methods and annotations were highlighted because incorrect interpretations in this step leads to severe bias. The number of participants, type of samples, number of annotators, and label information were summarized in a table to provide a reference for building engagement datasets.

Finally, in addressing the RQ3, we discuss machine learning-based methods have been applied to develop automatic engagement estimation approaches in the literature. We found that between 2010 and 2022, classic machine learning algorithms (including support vector machines (SVMs) and decision trees (DTs)) were used more in previous work. However, since 2019, the trend has moved to deep learning algorithms, especially convolutional neural network (CNN)- and recurrent neural network (RNN)-based algorithms.

### Limitations and remaining challenges

There is bias in the subjective determination of whether an article was aimed at education/learning settings. For example, some articles appear to be aimed at other purposes, such as therapy for children with autism Rudovic et al. (2018b) or human-robot interactions Ben-Youssef et al. (2019). However, the articles were included if the authors perceived that there was subtle information about a learning activity or the possibility that the proposed action could applied in the education process.

Moreover, the combination of a clear engagement definition, and suitable machine learning methods allows learners’ engagement during learning activities to be measured automatically, including human-human interactions, human-computer interactions and human-robot interactions. The estimation performance is especially promising for deep learning-based methods. However, the practicality of the implementation in real education settings is not discussed in this review. Therefore, the implication and application of these automatic engagement estimation methods should be addressed in future work to address various research questions, such as “How does engagement estimation improve learning outcomes?”, “What conditions and requirements are needed in automatic engagement estimation applications?”, and “In what learning settings can automatic estimation be applied?”.

Furthermore, we discuss several remaining challenges, including cognitive engagement, personalized engagement, and machine-learning pitfalls.

*Cognitive engagement* Table 2 shows that most automatic engagement research has focused on behavioural and emotional engagement and that affective data, especially appearance-based video data, were mostly utilized to estimate engagement. However, cognitive engagement, which can be determined through self-regulated learning or pre-post tests, plays an important role in successful distance learning. Similar to behavioural and emotional engagement, cognitive engagement can be measured using questionnaires (Li et al., 2021). However, few studies (Table 2) have considered this type of engagement. Therefore, we believe that more engagement cues for cognitive engagement should be developed in future automatic engagement estimation research.

*Personalized engagement* Various definitions of engagement have been constructed in the field of education. Although engagement can be divided into three types (i.e., behavioural, emotional, and cognitive engagement), conceptualizations of engagement sometimes include only one or two of the three types. All three types can be considered to determine engagement levels (Fredricks et al., 2004). To the best of our knowledge, no research has answered how these engagement types evolve and change over time. Therefore, whether the engagement cues may take different forms depending on the age range, gender, ethnicity, and education level of the participants is unknown.

Moreover, facial physiognomy differences between people with different ethnic backgrounds may result in various distributions of engagement levels (Rudovic et al., 2018a). Several automatic engagement estimations are targeted participants with specific cultures or backgrounds. For example, as shown by Libin and Libin (2004), a child’s background, including their cultural or psychological profile, needs to be considered when designing therapeutic strategies.

Network personalization can be achieved using demographic information (culture and gender), followed by individual network layers for each child (Rudovic et al., 2018b). However, it is unknown how engagement estimation results can be generalized in actual applications (Bosch et al., 2016). Thus, the user target must be defined, and the data must be collected from participants with the appropriate cultural background (for example, learners with autism spectrum conditions (ASCs) (Tincani et al., 2009; Conti et al., 2015)) to train the model (Rudovic et al., 2018a). Therefore, automatic engagement estimation, which considers individual differences, remains an open challenge.

*Machine learning pitfalls* Machine learning (ML) methods have been applied in various fields; however, reproducibility is an issue, as reviewed by Kapoor et al. (2022). The review examined 20 reviews across 17 research fields and found errors in 329 papers that used ML-based methods. While experienced machine learning practitioners are well aware of these errors, researchers in other disciplines may not be (DeepLearning.AI. 2022). Although education research was not included in the review (Kapoor and Narayanan 2022), we found similar issues (such as no training-testing splits, sampling biases, and pre-processing the training and test sets together) in the selected articles (see Appendix Table 4). The misuse of ML can generate invalid results that are irreproducible in implementations in real-world educational settings. Therefore, automatic engagement researchers should be aware of these issues (Kapoor and Narayanan 2022). Furthermore, education experts and ML experts could collaborate on engagement research to develop more effective models (DeepLearning.AI, 2022).

## Data Availability

Not applicable.

## Appendix

Tables 2, 3, and 4.

**Table 2:** An overview of the selected articles to address RQ1

Author	Journal	Domains				Engagement type			Engagement cues			
		HCI	HRI	A/H	Cls.	B.	E.	C.	Aff.	BE	PC	LF
(USTC-NVIE) Wang et al. (2010)	IEEE Transactions on Multimedia	$\checkmark$					$\circ$		$\bullet$			
Cocea et al. (2011)	IEEE Transactions on Learning Technologies	$\checkmark$				$\circ$						$\bullet$
AlZoubi et al. (2012)	IEEE Transactions on Affective Computing			A			$\circ$				$\bullet$	
S-Syun et al. (2012)	International Journal of Advanced Robotic Systems		$\checkmark$				$\circ$		$\bullet$			
Whitehill et al. (2014)	IEEE Transactions on Affective Computing	$\checkmark$					$\circ$		$\bullet$			
Schiavo et al. (2014)	Intelligenza Artificiale	$\checkmark$				$\circ$	$\circ$		$\bullet$			
Woo-Han Yun et al. (2015)	International Journal of Machine Learning and Computing	$\checkmark$					$\circ$		$\bullet$			
(DAiSEE) Gupta et al. (2016)	Conference proceedings	$\checkmark$					$\circ$		$\bullet$			
Zaletelj et al. (2017)	EURASIP Journal on Image and Video Processing				$\checkmark$	$\circ$			$\bullet$			
Monkaresi et al. (2017)	IEEE Transactions on Affective Computing	$\checkmark$					$\circ$		$\bullet$		$\bullet$	
(UE-HRI) Youssef et al. (2017)	Conference proceedings		$\checkmark$									
(BAUM-1) Zhalehpour et al. (2017)	IEEE Transactions on Affective Computing	$\checkmark$										
Hussain et al. (2018)	Computational Intelligence and Neuroscience	$\checkmark$				$\circ$		$\circ$				$\bullet$
Psaltis et al. (2018)	IEEE Transactions on Games	$\checkmark$					$\circ$		$\bullet$	$\bullet$		
Rudovic et al. (2018b)	SCIENCE ROBOTICS		$\checkmark$			$\circ$			$\bullet$		$\bullet$	
Ninaus et al. (2019)	Computers & Education	$\checkmark$					$\circ$			$\bullet$		
Yue et al. (2019)	IEEE Access		$\checkmark$			$\circ$	$\circ$	$\circ$	$\bullet$	$\bullet$		$\bullet$
(AffectNet) Mollahosseini et al. (2019)	IEEE Transactions on Affective Computing	$\checkmark$					$\circ$		$\bullet$			
Celiktutan et al. (2019)	IEEE Transactions on Affective Computing		$\checkmark$			$\circ$			$\bullet$		$\bullet$	
Youssef et al. (2019)	International Journal of Social Robotics		$\checkmark$				$\circ$		$\bullet$			
Olivetti et al. (2019)	Applied Science	$\checkmark$					$\circ$			$\bullet$		
Ashwin et al. (2020b)	Education and Information Technologies				$\checkmark$	$\circ$	$\circ$		$\bullet$			
Ashwin et al. (2020a)	Future Generation Computer Systems				$\checkmark$		$\circ$		$\bullet$	$\bullet$		
Pabba et al. (2022)	Expert Systems (Special Issues)				$\checkmark$		$\circ$		$\bullet$	$\bullet$		
Duchetto et al. (2020)	Frontiers in Robotics and AI		$\checkmark$				$\circ$		$\bullet$			
Yun et al. (2020)	IEEE Transactions on Affective Computing		$\checkmark$			$\circ$	$\circ$		$\bullet$			
Zhang et al. (2020)	Journal of Educational Computing Research	$\checkmark$				$\circ$	$\circ$		$\bullet$			$\bullet$
Liao et al. (2021)	Applied Intelligence	$\checkmark$					$\circ$		$\bullet$			
Li et al. (2021)	Computers & Education	$\checkmark$					$\circ$	$\circ$	$\bullet$			$\bullet$
Bhardwaj et al. (2021)	Computers & Electrical Engineering	$\checkmark$					$\circ$		$\bullet$	$\bullet$		
Goldberg et al. (2021)	Educational Psychology Review				$\checkmark$	$\circ$	$\circ$	$\circ$	$\bullet$			
Chatterjee et al. (2021)	Frontiers in Neuroscience			H			$\circ$				$\bullet$	
Youssef et al. (2021)	IEEE Transactions on Affective Computing		$\checkmark$			$\circ$			$\bullet$			
Sümer et al. (2021)	IEEE Transactions on Affective Computing				$\checkmark$		$\circ$		$\bullet$			
Trindade et al. (2021)	International Journal for Innovation Education and Research	$\checkmark$						$\circ$				$\bullet$
Ma et al. (2021)	International Journal of Information and Education Technology	$\checkmark$					$\circ$		$\bullet$			
Thiruthvanathan et al. (2021)	International Journal of Intelligent Engineering & Systems	$\checkmark$					$\circ$		$\bullet$			
Altuwairqi 2021 et al. (2021b)	Signal, Image and Video Processing	$\checkmark$				$\circ$	$\circ$		$\bullet$			$\bullet$
Vanneste et al. (2021)	Mathematics				$\checkmark$	$\circ$	$\circ$		$\bullet$			
Hasnine et al. (2021)	Procedia Computer Science	$\checkmark$					$\circ$			$\bullet$		
Delgado et al. (2021)	Conference proceedings	$\checkmark$					$\circ$		$\bullet$			
Engwall et al. (2022)	ACM Transactions on Human-Robot Interaction		$\checkmark$				$\circ$		$\bullet$	$\bullet$		
Mehta et al. (2022)	Applied Intelligence	$\checkmark$					$\circ$		$\bullet$			
Dubovi et al. (2022)	Computers & Education	$\checkmark$					$\circ$	$\circ$	$\bullet$		$\bullet$	
Thomas et al. (2022)	Computers and Education: Artificial Intelligence	$\checkmark$					$\circ$		$\bullet$			$\bullet$
Shen et al. (2022)	Multimedia Systems	$\checkmark$					$\circ$		$\bullet$	$\bullet$		
Apicella et al. (2022)	Scientific Reports	$\checkmark$					$\circ$	$\circ$			$\bullet$	

*HCI* human-computer interaction; *HRI*human-robot interaction; *A* embodied conversational Agent; *H* human-human interaction; *Cls.* classroom; *B.* behavior; *E.* emotional; *C.* cognitive; *Aff.* affective; *BE* basic emotions; *PC*physiological cues; *LF* log file (including log activity)

**Table 3:** An overview of the dataset in the selected articles to address RQ2

Dataset	Type	Setting	Stimuli	Participants	Samples	Annotators	Label	Publicity
(USTC-NVIE) Wang et al. (2010)	ER	S &P	3-4 mins emotional and 1-2 mins neutral videos from internet	215 healthy students (157 M and 58 F)	236 apex images. Visible and thermal.	5 EAs & self-report.	**6 basic emotions** (happiness, sadness, surprise, fear, anger, and disgust), average **arousal** and **valence**.	Yes^a^
Cocea et al. (2011)	E	WE	An online course (HTML-tutor) in 7 sessinos	48 users	14 logged events	3 EA	Engaged, disengaged, or neutral	N/A
AlZoubi et al. (2012)	E	S	An intelligent tutoring system with conversational dialogues (AutoTutor) in 45 mins learning session	27 students	20-second interval of biosensor signals	Retrospective self-report	8 affective states: boredom, confusion, curiosity, delight, flow/**engagement**, surprise, and neutral.	N/A
S-Syun et al. (2012)	ER	S	An intellingent robot platform (MERO): greeting, identification, and a questions game.	10 participants	1,400 elements	(humans numerical values)	4 facial expression states: smiling, surprised, neutral and angry	N/A
Whitehill et al. (2014)	E	S	A cognitive skills training software .	34 undergraduate students	10 seconds videos	7EA	Engaged, Not Engaged ([Very engaged,Engaged],[Nominally engaged,Not Engaged])	N/A
Schiavo et al. (2014)	E	S	A video game: single player level, “Operation 40” of “Call of Duty - Black Ops” video game.	22 participants (3 F, 19 M)	12420 samples	self-annotate using ExperienceSampling Method (ESM) (Larson and Csikszentmihalyi 2014)	Neutral, **Engaged**, Stress	N/A
Woo-Han Yun et al. (2015)	E	S	A testing interactive software.	12 Children	2,745 of 30 second video clips	1 EA	4 engagement levels: high/low interest, low/high boredom	N/A
(DAiSEE) Gupta et al. (2016)	E	W	2 videos (educational and recreational)	112 students (32 F and 64 M)	9,068 clips (10 secs)	10 EA	4 levels of 4 affective states: **engagement**, frustration, confusion, boredom	Yes^b^
Zaletelj et al. (2017)	E	S	4 lecturing sessions (@25-min) in offline classroom setting	18 students	videos and kinect features	5 EA	3-level scale attention score (high, medium, low)	N/A
Monkaresi et al. (2017)	E	S	Writing task (draft-feedback-review)	22 students	1,325 video segments	Concurrent and retrospective self-report	Not engaged, engaged	N/A
(UE-HRI) Youssef et al. (2017)	E	S	Interaction with Pepper robot	195 participants (125 M, 70 F)			Sign of Engagement Decrease (SED), Early sign of future engagement BreakDown (EBD), engagement BreakDown (BD), Temporary disengagement (TD)	Yes^c^
(BAUM-1) Zhalehpour et al. (2017)	ER	S &P	Short video clips	31 subjects (Turkish)	1,184 clips	5 EA	13 emotional and mental states, which are Anger (An), Disgust (Di), Fear (Fe), Happiness (Ha), Sadness(Sa), Surprise (Su), Boredom (Bo), Contempt (Co), Unsure (Un), Neutral (Ne), Thinking (Th), Concentrating (Con), Bothered (Bot)	Yes^d^
Hussain et al. (2018)	E	WE	Social science course on virtual learning environment (VLE)	383 students	Log file	N/A	IF (score on the assessment>= 90) OR (final results=Pass AND total number of clicks>- average clicks), then label = high engagement. Otherwise -> Low engagement	N/A
Psaltis et al. (2018)	ER	S &P	prosocial games: Path of Trust (original version and stripped-down version)	72 participants	750 videos from 15 subjects (3 seconds)	Retrospective self-reports	5 basic emotions (anger, fear, happines, sadness, surprise)	Yes^e^
Rudovic et al. (2018b)	E	S	25 min therapy session with NAO robot to learn four basic emotions: sadness, fear, anger, and happines	35 Chld.(17 from Japan, 18 from Serbia) ages 3 to 13 with autism	10s video fragment	5EA	6 engagement level [0-5] = evasive, non-compliance, indifferent, low engagement, mid engagement, high engagement	N/A
Ninaus et al. (2019)	ER	S	1) The number line estimation task, 2) watching a short clip.	122 participants	Image frames	Self-report	joy = “excited” or “inspired”, activity/interest = “attentive”, “active”, afraid = “distressed”, “scared”, upset = “irritable”, “hostile”	N/A
Yue et al. (2019)	ER	S &WE	MOOC course titled “Data Processing Using Python” with course 5=10 mins videos, teaching materials and quizzes.	46 participants	7224 learning performance instances	self-report and quiz score	7 emotions (Neutral, Happy, Disgust, Sad, Surprise, Fear, Anger),Eye Movement (writing, read, type), course: score	N/A
(AffectNet) Mollahosseini et al. (2019)	ER	W	Images collected from internet	450,000 subjects	Training set: 23,901 images Validation set: 3,500 images	12 EA for 450,000 images. 2EA for 36,000 images	’8 emotion categories (neutral, happy, ,sad, surprise, fear, disgust, anger, contempt), valence and arousal (continuous)	Yes
Celiktutan et al. (2019)	E	S	HHI: dyadic interactions, HRI: triadic interactions	18 students	290 clips of HHI, 456 clips of HRI, and 746 clips in total for each data modality (276 physiological clips of HHI)	self-report	Big Five personality traits (extroversion, neuroticsm, openness, agreeableness, conscienctiousness), 10-point likert scale of engagement	Yes^f^
Youssef et al. (2019)	E	S	Interaction with Pepper robot	278 users (182 M, 96 F)	209 interactions featuring a single user, and 69 multiparty interactions	2 EA using ELAN	Sign of Engagement Decrease (SED), engaged	Yes^g^
Olivetti et al. (2019)	E	S	A virtual learning environment (A European Enterpreneurship VET Model and Assessment)	12 participants (6 F, 6 M)	3D videos	2 EA and self-report	Engagement level 1,2,3	N/A
Ashwin et al. (2020b)	E	S &P	Offline classroom	50 students	24000 posed images of 50 students, 36000 images spontaneous	slef-anotate and 2 EA	**Engaged**, boredom and neutral	N/A
Ashwin et al. (2020a)	E	S &P	Offline classroom	350 students (Indian)	2900 posed images (1450 are multiple students in a single frame), 72000 spontaneous images	30 EA	Attentive (happiness, surprise, delight, **engaged**), in-attentive (sadness, fear, disgust, boredom, sleepy, frustrated, confused).	N/A
Pabba et al. (2022)	E	S	Offline classroom	50 (31 M and 19 F) (Indian)	1193 images (30 minutes)	5 EA	Engagement level academic affective states (low:Boredom, sleepy;Medium: Yawning, frustrated, confused;High: Focused)	N/A
Duchetto et al. (2020)	E	S		227 people (122 F, 105 M. 138 adults, 89 minors)	3,106 videos (10 fpr)	3 EA Using NOVA	Engagement score :High, low, medium	N/A
Yun et al. (2020)	E	S	Interactive multi-intelligence material	20 children (Asian)	356 video/images	3 and 7 EA	Engaged, Disengaged ([High Engagement, Low Engagement], [Low Disengagement, and High Disengagement]) [17:11:5:1]	N/A
Zhang et al. (2020)	E			47 students (28 M,19 F)	26 hours video (2 seconds image) and mouse movement	8 EA	1-5 engagement scale (but only 2 class classification Engaged, not engaged)	N/A
Liao et al. (2021)	Used Public Avilable dataset: Dataset for Affective States in E-Environments (DAiSEE)							
Li et al. (2021)	E-R	S	A virtual patient in BioWorld	61 medicalstudents	167 segments, videos (10 seconds)	self-report, 1 EA	8 clinical behaviors, 2 performances (shallow/surface, high/deep)	N/A
Bhardwaj et al. (2021)	E	S	Online class	1000 participants	Emotion scores	10 EA	0-5 scale engagement level and emotions: angry, disgust, fear, sad, surprise and neutral	Yes^h^
Goldberg et al. (2021)	E	S	offline classroom (90 mins), knowledge test	52 students (only 30 were used due to occlusions)	Videos	self-report and 6 EA using CARMA	-2 to +2 engagement scale:	
Chatterjee et al. (2021)	E	S	Dyadic conversation	16 dyads	Naturalistic conversations (15 minutes)	Self-report	Engagement level (none to very high), Engagement scale (0-100)	N/A
Youssef et al. (2021)	E	S	Interaction using Pepper robot	195 participants (70 F, 125 M)	124 interactions to feature a single user, 71 multiparty interactions (40 started as multiparty and ended as single user)	EA	Signs of User Engagement Breakdown (UEB): Breakdown, No Breakdown	N/A
Sumer et al. (2021)	E	S	Offline classroom	15 students	360 audio-visual recording	2 EA using CARMA every second	3 engagement class label (0,1,2)	N/A
Trindade et al. (2021)	ER	WE	Courses in Moodle		2752 Moodle record data from 2015-2019			N/A
Ma et al. (2021)	Used Public Avilable dataset: Dataset for Affective States in E-Environments (DAiSEE)							
Thiruthvanathan et al. (2021)		Used Public Avilable dataset: DAiSEE, iSAFE, ISED						
Altuwairqi 2021 et al. (2021b)	E	S	Writing task	42 participants	164 videos, mouse and keyboard log	Self-annotation	Strong, high, and medium engagements	N/A
Vanneste et al. (2021)	E	S	On lectures (hybrid virtual classroom)	14 students (4 F,10 M)	1031 clips (only 37-185 were annotated)	Self-report, EA	0,1,2 engagement	N/A
Hasnine et al. (2021)	E	S	Interactive lecture, lecture video taken from YouTube (28s)	11 students		N/A (concentration index (CI))	Highly-engaged, engaged, disengaged	
Delgado et al. (2021)	E	WE	Math problem on MathSpring.org	19 students	400 videos(18,721 frames)	3 EA	**Engaged** (looking at the screen or looking at their paper), wandering	N/A
Engwall et al. (2022)	E	S	Robot interaction (with Furhat anthropomorphic robotic head) in Wizard-of-Oz setup	33 language learners	50 audio-visual conversational videos (38 video recordings, 353 of 5s clips)	1 EA (audio recordings), 3 EA (video recordings), 9 EA (2s clips)	High and Low engagement. Clips (very dissengaged, dissengaged, neutral, engaged, very engaged)	N/A
Mehta et al. (2022)	Used Public Avilable dataset: Dataset for Affective States in E-Environments (DAiSEE)							
Dubovi et al. (2022)	ER	S	The Medication Administration Test (MAT), PANAS questionnaires	61 nursing students	Data streams, and pre-and post test context knowledge test	Self-report using PANAS	10 positive emotions and 10 negative emotions Positive and Negative Affect Scale (PANAS)(Watson et al. 1988)	N/A
Thomas et al. (2022)	Used Existed dataset^i^							
Shen et al. (2022)	Used Public Avilable dataset: JAFFE, CK+, RAF-DB							
Apicella et al. (2022)	E	S	Cognitive task (Continuous Performance Test), background music, social feedback	21 students	45 seconds acquisition EEG signals	Self-report, Performance index	High or low emotion engagement, high or low cognitive engagement	N/A

*ER* engagement-related dataset; *E* engagement dataset; *S* spontaneous; *P* posed; *W* in-the-Wild; *WE* web-based learning environment; *EA* external annotator;

^a^stated in the abstract but the database link is unavailable

^b^ https://people.iith.ac.in/vineethnb/resources/daisee/index.html

^c^ https://adasp.telecom-paris.fr/resources/2017-05-18-ue-hri/

^d^ https://archive.ics.uci.edu/ml/datasets/BAUM-1

^e^ https://vcl.iti.gr/masr-dataset

^f^ https://www.cl.cam.ac.uk/research/rainbow/projects/mhhri/

^g^ https://adasp.telecom-paris.fr/resources/2017-05-18-ue-hri/

^h^ Partially

^i^ IIITB Classroom Seminar dataset, IIITB Online Seminar dataset, IIITB Presentation style dataset, LectureVideoDB, ClassX, IIIT-AR-13K

**Table 4:** An overview of the method used in the selected articles to address RQ3

Author	Input device/modality	Input features	Estimation method	Performance metrics
Wang et al. (2010)	Thermal camera	Grayscale images pixels	**Feature extraction**: PCA, PCA + LDA, AAM, and AAM+LDA. **Classification**: KNN.**Validation**: LOOCV	Accuracy
Cocea et al. (2011)	Log file	30 log attributes	**WEKA**. 8 algorithms: 1) BNs, 2) LR, 3) simple logistic classification (SL), 4) Instance-based classification with Ibk algorithm (IBk), 5) Atrribute selected classification using J48 classifier and Best first search (ASC), 6) Bagging using REP (reduced error pruning) tree classifier (B), 7) Classification via Regression (CvR), 8) DTs	Accuracy (highest 91%)
AlZoubi et al. (2012)	3 sensors (electrocardiogram (ECG), facial electromyogram (EMG), galvanic skin response (GSR)), webcam, screen recorder.	117 features (EEG, currogator musle EMG, finger tips GSR)	**Preprocess**: low/high pass filter. **Feature extraction**: using Augsburg Biosignal Toolbox (Wagner et al.). **Classification**: PRTools 4.0 (de Ridder et al. 2017), a pattern recognition library for Matlab. 9 classifiers: 1) SVM with linear kernel (SVM1), 2). SVM with polynomial (SVM2), 3) KNN ($k=3$). 4) KNN ($k=5$), 5) KNN ($k=7$). 6) NB, 7) Linear Bayes Normal Classifier (LBNC), 8) Multinomial LR, 9) C4.5 DT. **Validation**: 10-fold cross validation with 20:7 train:test ratio	Kappa statistic and F1-scores. (KNN and LBNC yielded the best detection)
S-Syun et al. (2012)	Microphone, camera, Depth sensor	Oculesic, kinesic, proxemic, vocalic, person identity cue features	**Oculesic** (gaze direction), **Kinesic** (facial expression, movement, body posture/gesture), proxemic (body posture/gesture, spatial relation), vocalic (user call), person identity cue (Spatial relation, face identification). **Feature extraction**: OpenNI library. **Binary classifications** (inattention and attention): Fuzzy-based classification algorithm (FMMNN classifier). Fuzzy min-max neural networks (FMMNN) with 7 input nodes. **Validation**: 7:3 training:test samples	Accuracy 86%
Whitehill et al. (2014)	Camera	Facial features	**Feature extraction**: using CERT. **Binary Classification**: Boost (BF), SVM (Gabor), MLR (CERT). **Validation**: 4-fold cross-validation	2-alternative forced choice (2AFC)
Schiavo et al. (2014)	Camera	Head movement and face features	**Features extraction**: using face actions and expression recognition (Joho et al. 2011). **3-class classification**: SVM. **Validation**: LOOCV	Accuracy=73%, F-score = 63%
Woo-Han Yun et al. (2015)	Camera	55 features of face and head information	**Pre-processing**: median filtering and aggregation method (mean, median, max, min, standard deviation (STD), range, rate of zero crossings (ZCR). **4-class classification**: relevance vector classifier (RVC), a spare version of Bayesian kernel logistic regression or Gaussian process classification (GPC).	Accuracy = 78.53%, Balanced Accuracy = 70.64%
Gupta et al. (2016)	Camera	Image pixels	**Classification**: InceptionNet, C3D, LRCN.	Accuracy
Zaletelj et al. (2017)	Kinect one sensor	2D and 3D gaze point and body posture data	**3-class classification**: DT (simple and medium), KNN (coarse, medium, and weight), Bagged Trees, Subspace KNN	Accuracy = 75.3%
Monkaresi et al. (2017)	Kinect face tracker and ECG sensors (BIOPAC MP150 system)	kinect face tracker features, LBP-TOP, heart rate data	**Pre-process**: RELIEF-F for feature selection, Synthetic Minority Oversampling Technique (SMOTE) to handle the data imbalanced. **Classifications** using WEKA: Updateable NB, BN, LR, classification via cLustering, rotation forest, dagging. **Validation**: LOOCV.	AUC = 0.758 and 0.733.
Youssef et al. (2017)	No estimation method. Only proposed dataset.			
Zhalehpour et al. (2017)	Camera	Images	**Face tracking**: CHEHRA tracker. **Classification**: SVM. Accuracy: 5-class classification = 75.32%, 8-class = 65.84%	
Hussain et al. (2018)	Log file	Number of clicks and activity types	Activity types includes dataplus, forumng, glossary, oucollaborate, oucontent, resource, subpage, homepage, and URL. **Binary classification**: decision tree (DT), J48 (belongs to DT family), CART, JRIP decision rules, GBDT, NB. **Validation**: 10-fold cross validation	Accuracy, Recall, AUC, Kappa
Psaltis et al. (2018)	Kinect face tracker	Facial expression, Body motion features, average time of responsiveness.	Feature for emotional engagement: facial expression and body motion. Feature for behavioral engagement: average time of responsiveness. **Binary classification**: unimodal ANN classifiers. **Validation**: 4-fold validation. Testing on: three primary schools.	Accuracy = 85%
Rudovic et al. (2018b)	Audiovisual sensors from NAO robot and physiological sensors to provide heart-rate, electrodermal activity, body temperature, and accelerometer data.	Face, body, physiology features, CARS, the demographic features (culture and gender)	**Pre-process**: OpenFace, OpenPose openSMILE (Eyben et al. 2013), and self-built tools for feature extraction. DeepLift for feature selection. **Regression**: personalized perception of affect network (PPA-net) whis based on ANN and clustering using t-SNE.	Intra-class correlation (ICC) $= 65\% \pm 24$ (average ± SD)
Ninaus et al. (2019)	Webcam	Image frames	**Pre-process**: Microsoft’s Emotion-API classifying the prevalence of the 6 basic emotions for each frame of the captured videos (’fear’ and ’disgust’ are excluded to enhance the quality of the data). **Classification**: SVM ensembles using “classyfire” package in R statistical environment. Questionnaires were analyzed using separate multivariate ANOVAs	Accuracy $\approx 64.18\%$
Yue et al. (2019)	Microsoft LifeCam webcam and Tobii Eye Tracker 4C	Video/images, eye movement, and click stream data.	Fine-tuning parameters by transfer learning for CNN: VGG16, InceptionResNetv2. **Classification**: CNN and LSTM. **Regression**: CART, random forest, GBDT. **Validation**: 10-fold cross validation.	Accuracy = 76.08% for facial expressions recognition, 81% for eye movement behavior. R2 metric = 0.98 ofof course performance prediction.
Mollahosseini et al. (2019)	N/A	Images	CNN (AlexNet) and SVR on Valnce and Arousal labels	RMSE, CORR, SAGR, CCC.
Celiktutan et al. (2019)	Cameras (2 static & 2 dynamic), 2 biosensors	Image, sensor data	**Binary classifictions**: SVMs. **Validation**: a double LOOCV.	
Youssef et al. (2019)	Robot’s camera	Distance; head, gaze and face streams; speech;looking and listening.	**Feature extraction**: OpenFace and Pepper OKAO software. **Binary classification**: LR, DNN, GRU, LSTM. **Validation**: 3-fold cross validation	Accuracy, F1-Score, AUC
Olivetti et al. (2019)	Camera	Images (geometrical description)	**3-class classification**: SVM	The classification result was compared with the questionnaire.
Ashwin et al. (2020b)	Camera	299x299x3 image with RGB with facial expressions, hand gestures and body postures present	**Pre-processing** = data augmentation. **Classification**: transfer learning with inception v3. **Hybrid CNN** = CNN-1 + CNN-2. CNN-1 for single student in as single image frame. CNN-2 for multiple students in a single image frame. **Validation**: 10-fold cross validation	**Posed**: accuracy = 86%, recall = 89%, precision = 91%, F1-score = 84%, AUC = 90%. **Spontaneous**: accuracy = 70%, recall = 72%, precision = 77%, F1-score = 62%, AUC = 69%
Ashwin et al. (2020a)	Camera	Images with facial expressions, hand gestures and body postures present	**Classification**: CNN with pre-trained on GoogleNet architecture(Krizhevsky et al. 2017). **Validation**: 10-fold cross validation.	Accuracy = 76%
Pabba et al. (2022)	Camera	48x48 image pixels	Add additional public dataset: BAUM-1,DAiSEE, and Yawning Detection Dataset (YawDD)^a^ . **Pre-process**: face and head detection (using multi-task cascade CNN (MTCNN)), face alignment, data augmentation. **6-class classification**: CNN.	Accuracy = 76.9%
Duchetto et al. (2020)	Head camera of the robot	RGB frame-by-frame image	**Face detection**: CNN. **Regression**: LSTM. Build the model using TOGURO dataset and evaluated on UE-HRI.	AUC=0.89
Yun et al. (2020)	Camera, Kinect V2	Facial features	**Classification**: CNN with fine tunning by using a pre-trained network (VGG-3D model). **Validation**: 6-fold cross-validation, leave-one-labeler-out cross-validation (LOLOCV).	Accuracy, AUC of ROC (ROC), AUC of PRs (PRs), MCC, F1-score, balanced accuracy, specificity (true positive and negative rate).
Zhang et al. (2020)	Camera	grayscale image (100 x 100 pixel)	**Feature extraction**: adaptive weighted LGCP. **Binary classification**: fast sparse representation (AWLGCP &FSR). **Validation**: 10-fold validation. Compare: the four methods (CLBP-SRC, Gabor-SVM, active shape model-SVM, and AWLGCP &FSR).	
Liao et al. (2021)	N/A	DAiSEE and EmotiW images	**Face detection**: MTCNN. **Pre-process**: resize images to 224$\times$224 and pre-trained on VGGFace2. **4-class classification** and **regression**: Deep Facial Spatiotemporal Network (DFSTN) = pretrained SE-ResNet-50 (SENet) for extracting facial spatial features, and LSTM Network with Global Attention (GALN). **Validation**: 5-fold cross-validation.	Accuracy = 58.84% and MSE = 0.0422 on DAiSEE. MSE = 0.0736 on EmotiW.
Li et al. (2021)	Camera, log file	Facial features (Gaze, Pose, FAU) and 8 clinical behaviors	**Performance (correcteness)** labelling: for problem solving process (Measure cognitive engagement). **Feature extraction**: using OpenFace. Calculate mean and std of each facial features. **Feature selection**: recursive feature elimination random forest (RFE-RF). **Binary classification**: NB, KNN, DT, RF, SVM. **Validation**: 10-fold-cv for feature selection. Use students self-reports of cognitive engagement states as the ground-truth	
Bhardwaj et al. (2021)	FER-2013 dataset (image), and MES dataset	images	**Face detection**: OpenCV. **Binary classification**: CNN. First, calculating weights matrix of emotions, then calculating MES and detecting engagement.	
Goldberg et al. (2021)	3 Cameras	Eye gaze, head pose, and facial expressions.	**Feature extraction**: OpenFace. **Regression**: *Model 1*: multiple linear regression. *Model 2*: two additional linear regression. *Model 3*: add learning prerequisites.	MSE= 0.05. Pearson correclation coefficient between manual annotations’ mean level and prediction models *r* = .70 , *p* = 0
Chatterjee et al. (2021)	electrocardiography, skin conductance, respiration, skin temperature, Yeti X microphone, webcams	Electrocardiography, skin conductance, respiration, skin temperature signals	**Pre-process**: lowpass/highpass filter using MATLAB/Simulink. **Regression**: a binary decision tree, leastsquares boosting, and random forest implemented in MATLAB 2020b. **Validation**: LOOCV	
Youssef et al. (2021)	Robot’s camera	Distance; head, gaze and face streams; Speech;Laser	Face detection: NAOqi People Perception.**Face extraction**: OKAO Vision sofware. Imbalanced issue: undersampling “No breakdown”, oversampling “Breakdown” class using SMOTE. **Binary classification**: LR, LDA, RF, and MLP. **Validation**: 5-fold cross validation.	AUC $\approx$ 0.72
Sümer et al. (2021)	Camera	Face features, head pose (without facial landmarks)	**Face detection**: RetinaFace. Multi channel settings : training Attention-Net for head pose estimation and Affect-Net for facial expression recognition CNN. **Pre-Process**: : PCA (for SVM). **3-class classification**: SVM (use majority voting), RF, MLP, LSTM with fine tunning (transfer learning) with AffectNet for facial expression and Attention-Net (300W-LP) for head pose with ResNet-50. **Tested** using different fusion strategies using RF engagement classifiers. Use of self-supervision and representation learning on unlabelled classroom data.	AUC = 0.84 (with personalization). Attention-Net better than Affect, given that the criteria for the manual annotation of engagement is not directly related to gaze direction or facial expression.
Trindade et al. (2021)	Log file	Teacher and students attributes	**WEKA**. Random Forest generated the best result.	AUC
Ma et al. (2021)	Use DAiSEE	Eye gaze, facial action unit, head pose (117 dimensions); and body pose (60 dimensions)	**Feature extraction**: OpenFace 2.0. **Pre-process**: 640x640 resolution at 10fps. **Feature Fusion**: Neural Turing Machine (NTM) architecture, which contains two basic components: a neural network controller and a memory bank. NTM workflow: read heads and write heads.	Accuracy = 60.2%
Thiruthvanathan et al. (2021)	Indian origin faces datasets DAiSEE, iSAFE, ISED	508 images from ISED and iSAFE, 5295 images from DAiSEE.	**Feature extraction**: light weight ResNet. **Classification**: ResNet classifier (CNN with 50 layers deep).	Accuracy, Precision, Recall, Sensitivity, Specificity and F1 score
Altuwairqi 2021 et al. (2021b)	Camera, mouse, keyboard behaviour	Key frame facial expressions.	Transfer learning using FER2013 and real-world affective faces (RAF). **3-class classification**: Naive Bayes (NB) classifier.	Accuracy and MSE.
Vanneste et al. (2021)	Camera	Upper body keypoints, eye gaze direction	**Feature for individual classification**: upper body keypoints (from 2s clips), **for collective classifications**: eye gaze direction. **Classification**: i3D model (CNN based) (Carreira and Zisserman 2017). **Multilevel regression**: to investigate how the engagement cues relate to the engagement scores. Calculate the CST (collective state transition) to measure classroom engagement.	Recall and Precision. Hand-raising and note-taking are not related to students individual self-reported engagement scores.
Hasnine et al. (2021)	Camera	Video	**Face detection**: Dlib. **3-class classification**: training with FER2013, then calculate the concentration index (CI) based on eye gaze and emotion weights. CI = (Emotion Weight x Gaze Weight) / 4.5	Accuracy = 68%
Delgado et al. (2021)	Camera	Images	**Classification**: utilizing CNN family including MobileNet (Mobilenets: Efficient convolutional neural networks for mobile vision applications), VGG (Very deep convolutional network for large-scale image recognition), Xception: Deep learning with depth-wise separable convolutions.	
Engwall et al. (2022)	Cameras and microphone	Audio and visual features	**Feature extraction**: OpenFace 2.0. **Feature selection**: verbal classifications using bag-of-words representations, accoustic-based classification,video-based classification. **Engagement classification through acoustic and visual**: classification using SVM, DT, Conditional Random Fields, KNN, HMM, Gaussian model, BN, and ANN. **Engagement classification through vocal arousal**: bidirectional LSTM network Speech Emotion Recognition implementation in the Matlab Deep Learning Toolbox. *Output*: anger and happines = High, neutral = Neutral, boredom and sadness = Low. **Engagement classification through face expression**: two SVM with linear and radial basis function (RBF) as kernel.	Listener engagement classification reached 65% balanced accuracy
Mehta et al. (2022)	Use DAiSEE and Emoti-W dataset	Images	**Pre-processing**: Dlib face detector. **4-class classification and regression**: 3D CNN with self-attention module, which enhances the discovery of new patterns in data by allowing models to learn deeper correlations between spatial or temporal dependencies between any two points in the input feature maps.	Classification accuracy = 63.59% on DAiSEE, regression MSE = 0.0347 on DAiSEE and 0.0877 = Emoti-W
Dubovi et al. (2022)	Eye tracker, EDA wearable wristband sensor, and webcam	Facial expression, eye-tracking, and EDA data	The stream data was collected and analysed using iMotion 9.0 with 7 basic emotions annotation (joy, anger, surprise, contempt, fear, sadness, and disgust). **Emotional engagement**: a Linear Mixed Effects Model (LMM) was established to estimate the self-reported changes in the PANAS self-report. **Cognitive engagement**: ANOVA was performed to assess the eye-tracking metrics differences.	
Thomas et al. (2022)	Use existed dataset^b^	Visual and verbal features	**Pre-process**: slide area and figure detection using RetinaNet, unique slide detection using Siamese network, text detection using Character-Region Awareness For Text detection (CRAFT) model. **Prediction**: pretrained with pretrained VGG-16 network. Supervised: LR with three classes (visual, verbal, or balanced). Unsupervised: clustering model with two clusters (visual, verbal). **Binary lassification**: sequential modeling using Temporal Convolutional Network (TCN) pre-trained with Micro-Macro-Motion (MIMAMO) Net model (Deng et al. 2020).	**At the segment level**: accuracy = 76%, F1-score = 0.82, MSE = 0.04. **At video level (binary classification: engaged/distracted)**: accuracy = 95%, F1-score = 0.97, MSE = 0.15
Shen et al. (2022)	Use JAFFE, CK+, RAF-DB dataset	Images	**Pre-process**: MK-MMD to calculate the distribution distance between the extracted features. **Transfer learning**: Domain adaptation technique was used to explore the additional facial images. Imbalanced issue: undersampling, and data augmentation. **4-class classification**: lightweight attention convolutional network for face expression recognition. Soft attention module (SE) was adopted to reduce the impact of the complex background.	Accuracy = 56%
Apicella et al. (2022)	EEG	EEG Signal	**Pipeline**: Filter bank, Common Spatial Pattern, SVM. **Pre-process**: artifact removal using indpendent componenet analysis (ICA), namely Runica module of the EEGLab tool. **Feature extraction**: 12-component Filter Bank. Imbalanced problem: Stratified leace-2-trials out. **Binary classification**: SVM, Linear Discriminant Analysis (LDA), KNN, shallow ANN, DNN, CNN (pre-trained in Common spatial pattern (CSP)).	SVM achieved the highest score accuracy = 76.9% for cognitive engagement, and 76.7% for emotional engagement.

*PCA* principle component analysis; *LDA* linear discriminant analysis; *AAM* active appearance model; *LOOCV* leave-one-subject-out cross validation; *KNN* K-nearest neighbors; *BNs* Bayesian Nets;*LR* logistic regression; *DTs* Decision Trees; *NB* Naive Bayes; *LBP-TOP* three orthogonal planes; **GBDT** gradient boosting trees; *CART* classification and regression tree; *CARS* - childhood autism rating scale; *GRU* - gated recurrent unit; *LSTM* long-short term memory; *CNN* convolutional neural network; *KNN* K-nearest neighbors; *BNs* Bayesian Nets; *LR* logistic regression; *DTs* - Decision Trees; *NB* Naive Bayes

*LOOCV* leave-one-subject-out cross validation; *LR* logistic regression; *RF* - random forest; *LDA* linear discriminant analysis; *MLP* Multi-layer Perceptron; *DT* decision tree; *BN* Bayesian Network; *HMM* -Hiden Markov Models; *LSTM* long short time memory; *EDA* electrodermal activity; *MK-MMD* Kernel Maximum Mean Discrepancies

^a^ https://dx.doi.org/10.21227/e1qm-hb90

^b^ ClassX, LectureVideoDB, IIIT-AR-13K, IIITB Online Lecture, IIITB Classroom Lecture dataset

## Footnotes

1 A natural visible and infrared facial expression database for expression recognition and emotion inference.

2 User engagement in spontaneous human-robot interactions.

3 Dataset for affective states in E-environment.

4 Multimodal human-human-robot interactions dataset for studying personality and engagement.

5 https://opencv.org/.
